# ENPP1 in Blood and Bone: Skeletal and Soft Tissue Diseases Induced by ENPP1 Deficiency

**DOI:** 10.1146/annurev-pathmechdis-051222-121126

**Published:** 2023-10-23

**Authors:** Carlos R. Ferreira, Thomas O. Carpenter, Demetrios T. Braddock

**Affiliations:** 1 Metabolic Medicine Branch, National Human Genome Research Institute, National Institutes of Health, Bethesda, Maryland, USA; 2 Department of Pediatrics, Yale University School of Medicine, New Haven, Connecticut, USA; 3 Department of Pathology, Yale University School of Medicine, New Haven, Connecticut, USA

**Keywords:** ectonucleotide pyrophosphatase/phosphodiesterase 1, ENPP1 deficiency, generalized arterial calcification of infancy, GACI, autosomal recessive hypophosphatemic rickets type 2, ARHR2, enthesopathy, pseudoxanthoma elasticum, PXE, ossification of the posterior longitudinal ligament, OPLL, dystrophic idiopathic spinal hyperostosis, DISH

## Abstract

The enzyme ectonucleotide pyrophosphatase/phosphodiesterase 1 (*ENPP1*) codes for a type 2 transmembrane glycoprotein that hydrolyzes extracellular ATP to generate pyrophosphate (PP_i_) and adenosine monophosphate, thereby contributing to downstream purinergic signaling pathways. The clinical phenotypes induced by ENPP1 deficiency are seemingly contradictory and include early-onset osteoporosis in middle-aged adults and life-threatening vascular calcifications in the large arteries of infants with generalized arterial calcification of infancy. The progressive overmineralization of soft tissue and concurrent undermineralization of skeleton also occur in the general medical population, where it is referred to as paradoxical mineralization to highlight the confusing pathophysiology. This review summarizes the clinical presentation and pathophysiology of paradoxical mineralization unveiled by ENPP1 deficiency and the bench-to-bedside development of a novel ENPP1 biologics designed to treat mineralization disorders in the rare disease and general medical population.

## 1. EXTRACELLULAR PURINERGIC METABOLISM

Ectonucleotide pyrophosphatase/phosphodiesterase 1 (ENPP1) is a type II transmembrane protein composed of four structurally distinct domains—two somatomedin B-like domains, a catalytic domain, and a nuclease domain. The catalytic domain lies in the extracellular space, where it cleaves high-energy phosphate bonds within nucleotide triphosphate compounds to generate extracellular pyrophosphate (PP_i_) and nucleotide monophosphates (1) or cyclic nucleotides such as 2′3′-cGAMP (cyclic guanosine monophosphate–adenosine monophosphate) to generate 5′-AMP and 5′GMP (2). The enzymatic products of ENPP1 catalysis generate extracellular signaling molecules that regulate whole organismal physiology—either directly or as ligands to cell surface or intracellular receptors. ENPP1 regulates soft tissue mineralization through generation of PP_i_, which inhibits extraskeletal calcification by incorporation into the hydroxyapatite crystal and elimination of crystal contacts necessary for crystal growth (3, 4). ENPP1 also regulates vascular endothelial proliferation through activity of AMP on purinergic endothelial cell receptors (5), and ENPP1 regulates the innate immune response by modulating inflammatory cytokines and the type I interferon response through hydrolysis of 2′3′-cGAMP—a ligand for the germline-encoded pattern recognition receptor STING (6, 7). This review focuses on diseases induced by ENPP1 loss of function or missense pathogenic variants, which induce mineralization phenotypes in affected patients that include life-threatening vascular calcifications in infants with generalized arterial calcification of infancy (GACI), severe phosphate wasting rickets in children with autosomal recessive hyperphosphatemic rickets type 2 (ARHR2), early-onset osteoporosis in middle adults, and perispinal ossifications and enthesopathies in a subset of patients diagnosed with ossification of the posterior longitudinal ligament (OPLL) and diffuse idiopathic skeletal hyperostosis (DISH).

**Figure 1:**
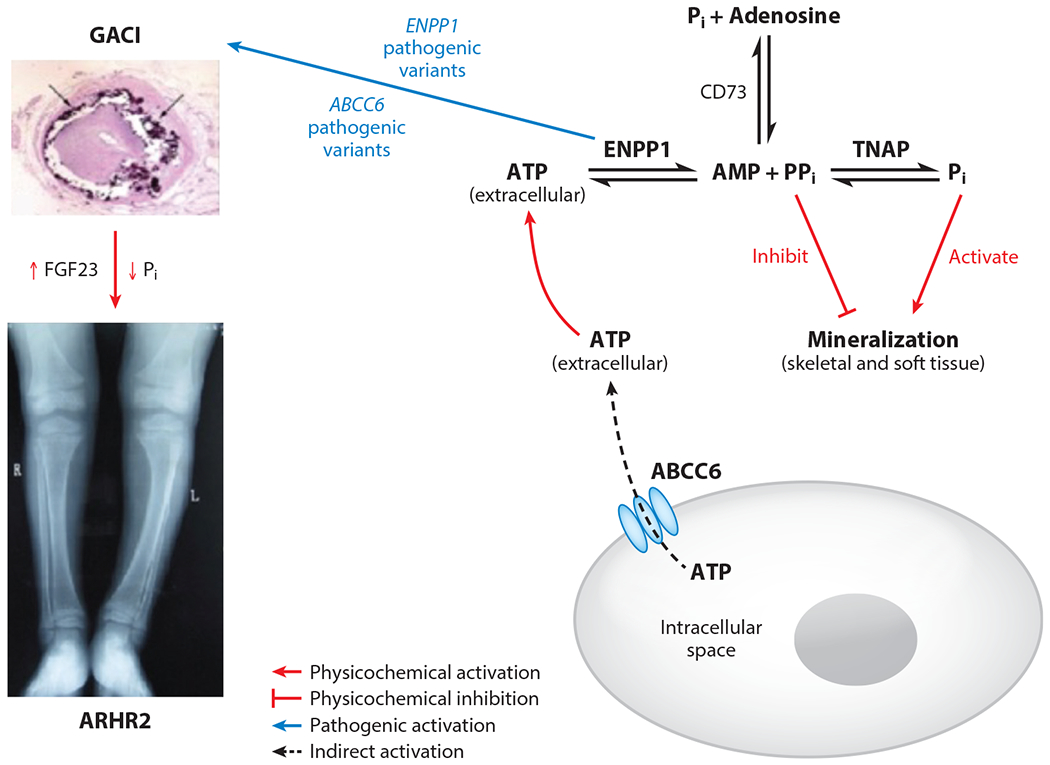
Metabolic diseases due to homozygous defects in extracellular purinergic metabolism. The liver-associated transmembrane transporter ABCC6 [adenosine triphosphate (ATP)-binding cassette subfamily C member 6] exports ATP into the extracellular space, which is then metabolized by ectonucleotide pyrophosphatase/phosphodiesterase 1 (ENPP1) into adenosine monophosphate (AMP) and pyrophosphate (PP_i_). AMP is metabolized into adenosine and phosphate (P_i_) by 5′-nucleotidase (CD73), and PP_i_ is metabolized into P_i_ by tissue-nonspecific alkaline phosphatase (TNAP). P_i_ promotes, and PP_i_ inhibits, hydroxyapatite, which constitutes the hard matrix of bone and ectopic calcifications in soft tissue. Pathogenic variants in *ENPP1* and *ABCC6* reduce extracellular plasma (PP_i_) to 10% and 30% of normal, respectively, inducing generalized calcification of infancy (GACI), in which approximately 50% of afflicted infants will succumb to life-threatening aortic and arterial calcifications. Those that survive will almost invariably exhibit phosphate wasting rickets in childhood, called autosomal recessive hypophosphatemic rickets type 2 (ARHR2).

To understand how a single enzyme induces such disparate clinical phenotypes, it is best to review the metabolic disorders induced by disruptions in the extracellular purine metabolic pathway (Figure 1). A liver-associated transmembrane transporter known as ATP-binding cassette subfamily C member 6 (ABCC6) mediates the export of ATP into the blood, where it is cleaved by ENPP1 into AMP and PP_i_. Tissue-nonspecific alkaline phosphatase cleaves PP_i_ into phosphate (P_i_), and 5′-nucleotidase (CD73) cleaves AMP into adenosine and phosphate. Phosphate complexes with calcium to form hydroxyapatite, which constitutes the hard mineral phase of bone as well as the extracellular mineral deposits seen in vascular calcification and the joint calcifications observed in patients with osteoarthritis. Pyrophosphate is able to replace phosphate in the growing hydroxyapatite crystals and thus renders the crystal contacts of the growing crystal lattice inaccessible, effectively preventing crystal growth; extracellular AMP and adenosine mediate vascular tone by regulating pre- and postglomerular vascular resistances, glomerular filtration rate, renin release, epithelial transport, intrarenal inflammation, and growth of mesangial and vascular smooth muscle cells (8). Remarkably, inactivating variants in several of the transporters and enzymes involved in extracellular purinergic metabolism results in mineralization disorders (Table 1). Pathogenic variants in ABCC6 induce reduced extracellular ATP leading to decreased plasma PP_i_, with a subsequent increase in hydroxyapatite deposition in nucleation sites near elastic fibers in the skin and eye, leading to a mineralization disorder called pseudoxanthoma elasticum (PXE). PXE patients suffer from skin calcifications, retinal findings including bleeding, and slowly progressive vascular calcifications (9). Pathogenic variants in ENPP1 result in markedly decreased plasma PP_i_ levels, leading to vascular calcifications as early as the second trimester of development in the life-threatening disease GACI (10). These children often present within the first week of life with stroke, severe hypertension and cardiac failure, and approximately 50% mortality by 6 months of age. Infants fortunate enough to survive will typically develop phosphate wasting rickets (ARHR2) (11, 12) in infancy as well as enthesopathies in their extremities and spines, which progressively worsen with time. Infants affected by pathogenic variants in tissue-nonspecific alkaline phosphatase suffer from the opposite problem—they are unable to mineralize their skeleton due to an excess in extracellular PP_i_ and reduced P_i_ in a disease known as hypophosphatasia (HPP) (13). Infants affected with the most severe forms of HPP die from hypoxia at birth because they are unable to expand their ribcage due to the severely deficient skeletal mineralization. Finally, pathogenic variants in CD73 induce calcifications in the arteries in the lower extremities as well as periarticular calcifications in the joints of the extremities in a disease known as arterial calcification due to CD73 deficiency (14). To understand the pathophysiology of these disorders we begin by examining the clinical phenotype and presentation of GACI infants, expand into the skeletal mineralization disorders associated with ENPP1 deficiency, and conclude with a discussion of the development of novel therapeutics for patients with ENPP1 deficiency and their application to mineralization disorders affecting the general medical population.

## 2. GACI

The first patient reported to have what we now call GACI was born in 1898 and reported in 1899 as a case of congenital atheroma of the aorta and pulmonary artery (15). The first patient in the English literature was reported in 1901, in an infant with arterial calcification and intimal proliferation (obliterative endarteritis) (16). A low concentration of plasma pyrophosphate (0.6 μmol/L; normal, 1–6 μmol/L) was detected in an infant in 1990 by Dr. Graham Stuart, a cardiologist in the Freeman Hospital at Newcastle (17). This child presented within a few hours of birth with poor feeding, cyanosis, heart failure, lethargy, and respiratory distress. Echocardiogram studies revealed calcifications in the pulmonary and aortic valves. Pulmonary artery calcifications were seen on fluoroscopic examination and calcification was evident in the internal elastic lamina of the posterior tibial artery on biopsy. A provisional diagnosis of idiopathic infantile arterial calcification, now known as GACI, was made, whereupon Dr. Stuart sent a plasma sample from the infant to John Kanis, an investigator at the University of Sheffield, for measurement of plasma PP_i_, an assay that was not routinely available and notoriously difficult to perform.

Dr. Stuart likely requested this test due to the association between PP_i_ and spontaneous mineralization reported in the early 1960s by Graham Russell and Herbie Fleisch, who had described the spontaneous formation of hydroxyapatite crystals in buffered solutions possessing physiologic concentrations of calcium and phosphate but not in the presence of plasma (3). The observations demonstrated calcium and phosphate to be at saturating concentrations in human plasma but nevertheless they did not spontaneously form hydroxyapatite crystals in plasma, demonstrating the presence of unidentified mineralization inhibitors in blood. They sought to purify these inhibitors out of urine, reasoning that renal stones would develop in their absence, identifying plasma pyrophosphate as a mineralization inhibitor capable of preventing spontaneous hydroxyapatite formation (18, 19).

After identifying a low plasma pyrophosphate level, Dr. Stuart administered a bisphosphonate, disodium etidronate, at a dose of 20 mg three times a day to the infant in view of a report attributing etidronate with survival beyond infancy to the drug. This prior report documented suppression and reversal of arterial calcifications and attributed the response to etidronate treatment (20). Nevertheless, Dr. Stuart’s patient succumbed to cardiac arrest at 8 weeks of age. Although Dr. Stuart’s testing and observations suggested low plasma pyrophosphate as the pathogenic mechanism leading to vascular calcifications in GACI, the failure of etidronate to prevent mortality in this patient [and his similarly treated sibling who died 5 years later (17)] led him to demur on a definitive disease mechanism, speculating instead that inherited disorders of iron metabolism and elastin structure might also be involved (21).

### 2.1. Clinical Presentation

GACI is the most severe form of ENPP1 deficiency and presents with widespread calcification and intimal thickening of large- to medium-sized vessels. The calcification is not intimal but occurs along the internal elastic lamina, separating the media from the intima. The proliferation of the intima leads to luminal narrowing, with subsequent ischemia. Spontaneous resolution of calcification has been reported in several individuals (22, 23). In those without continued evidence of calcification, arterial stenoses associated with intimal thickening have been observed (24, 25). Generalized arterial stenoses without prior evidence of arterial calcification have also been reported (26, 27). Thus, it appears that the two phenomena of arterial calcification and stenosis are independent from each other, and while calcification can spontaneously regress with time, the stenosis seems to remain.

Arterial calcification is detected in 77% of affected individuals, involving in decreasing order the aorta (68%), pulmonary arteries (52%), renal arteries (49%), coronary arteries (48%), carotid arteries (43%), mesenteric arteries (34%), femoral arteries (25%), and cerebral arteries (15%) (28). Arterial stenosis is present in at least 72.4% of GACI cases (5). Functional cardiovascular complications are seen in 58% of patients (28). There is a high risk of mortality within the first 6 months of life, which stabilizes with increasing age. The probability of mortality from GACI—independent of its underlying etiology—at 6 months is 50.6%, while the same risk for individuals with ENPP1 deficiency specifically is 33.5% (28). There are various explanations for this critical period in the first 6 months of life, followed by a later period of stability. First, in most cases the calcifications can regress. Second, even though patients can have arterial stenosis in the presence or absence of calcifications, arteries continue to grow in diameter with age as documented by serial imaging, although they are still reduced in caliber. According to Poiseuille’s law, the flow through a vessel is directly proportional to the fourth power of the radius. Thus, even a small linear growth in the diameter of an artery will lead to an exponential increase in its blood flow, so that slight increases in radius can lead to dramatic increases in blood flow.

### 2.2. Periarticular Calcifications in GACI

Almost another decade passed before Dr. Frank Rutsch treated an infant with tachypnea, cyanosis, and severe metabolic lactic acidosis in Dortmund, Germany (10). On the fifth day of life, the infant was placed on mechanical ventilation due to congestive heart failure and recovered after 19 days. Screening for metabolic disorders revealed only a carnitine deficiency, and the patient was treated with digoxin, furosemide, and spironolactone but additionally developed arterial hypertension at 6 months of age. Cardiac catheterization revealed a homogeneously hypertrophied left ventricle, a patent ductus arteriosus, hypoperfusion of the upper lobe of the left lung, and bilateral renal arterial stenoses. After the ductus arteriosus was addressed, the patient was treated with phentolamine and captopril but 8 months later developed painful swelling of the left wrist and ankle due to bilateral periarticular calcifications in the carpal and ankle joints. Biopsy of the left temporal artery demonstrated calcifications in the medial wall, and low pyrophosphate levels were reported in spot urine samples, ranging from 0.5 to 6.3 μmol/mmol creatinine (normal, 7.7–56.7 μmol/mmol creatinine). The diagnosis of GACI was therefore rendered, followed by radiographic and ultrasound identification of calcifications in the aorta, carotid, and celiac arteries. This patient was started on etidronate at 10 months of age and survived into adolescence.

At around this time, a rheumatologist at the University of California, San Diego, Dr. Terkeltaub, was examining the joints and synovial fluid in an Enpp1-deficient mouse called tiptoe walking (*ttw*) (29). *Ttw* mice were initially reported in 1981 as the product of brother-sister mating of the ICR mouse strain (30) and were found to have increased calcium deposition in their cartilage and ligaments (31) resulting in a severe myelopathy simulating aspects of the human disease OPLL (32), as well as osteopenia in their long bones when compared with wild-type (WT) littermates (33, 34). *Ttw* mice were eventually found to possess a nonsense variant (Gly568stop) in *Enpp1*, which severely limited the enzyme’s expression on cell surfaces and thus markedly reduced plasma PP_i_ levels (32). Importantly, the spinal hyperostotic and osteopenic murine phenotypes present in *ttw* mice heralded human phenotypes of ENPP1 deficiency, described further below.

Dr. Rutsch joined Dr. Terkeltaub’s lab after treating a GACI infant, and, together, they determined that the PP_i_ deficiency in GACI patients was due to low or absent levels of ENPP1 and that the loss of ENPP1 catalytic activity could not be compensated by any of the other members of the ENPP family known at the time (10). Indeed, structural and biochemical studies over the intervening two decades have found that despite nearly identical structures in the enzymatic active sites of the seven ENPP1 family members (35–40), only ENPP1 can generate extracellular pyrophosphate as evidenced by the nearly absent levels of plasma PP_i_ in patients with ENPP1 loss-of-function variants. The lack of redundancy among the ENPP family in PP_i_ generation accounts for the dramatic phenotypic consequences of ENPP1 deficiency in humans.

### 2.3. Current Therapeutic Strategies for GACI

Since ectopic calcification is a consequence of PP_i_ deficiency, one theoretical therapeutic option would be to rely on the administration of PP_i_. However, this approach is impractical given the short half-life of parenterally administered PP_i_, estimated to be approximately 30 min in a rat (41); the P-O-P skeleton of PP_i_ renders it hydrolysable by enzymes such as alkaline phosphatase. It has been known since the 1960s that PP_i_ prevents hydroxyapatite deposition (3), and studies in the 1960s and 1970s demonstrated that bisphosphonates, nonhydrolysable analogs of PP_i_ with a P-C-P backbone, inhibit the transformation of amorphous calcium phosphate into hydroxyapatite crystals (42, 43), the aggregation of calcium phosphate crystals (44), and, more specifically, the aggregation of hydroxyapatite crystals (45). As detailed above, in 1978 the vascular mineral deposition in GACI was identified as hydroxyapatite, leading to the use of bisphosphonates to attempt to inhibit the life-threatening vascular calcifications in neonates (20). Subsequently, 65% survival was reported in GACI patients receiving bisphosphonates compared with 31% of patients who were not treated (*p* = 0.026) (46). However, spontaneous regression of vascular calcifications in GACI patients in the absence of any therapy was later recognized (22, 23), calling into question the clinical relevance of these findings. A later study, using a start-time matched analysis to account for different ages of initiation of bisphosphonates over the critical period of high mortality, found a trend toward survival benefit that did not reach statistical significance. However, the confidence intervals did not overlap during the first month of life, indicating a possible survival benefit if bisphosphonates are initiated early. In fact, a survival analysis of bisphosphonates initiated within 6 months versus no bisphosphonates found a significant difference if bisphosphonates were initiated within the first week of life, but no survival benefit if initiated thereafter (28).

Newer bisphosphonates potently inhibit osteoclasts due to intracellular biological mechanisms so that relatively small amounts are administered to achieve their osteoclast inhibitory role, thus limiting the physicochemical effect seen with higher doses intended to prevent hydroxyapatite deposition. On the other hand, much larger and more frequent doses of etidronate, a first-generation bisphosphonate, can be administered at higher doses (≈20 mg/kg/day). In fact, etidronate was shown to be more potent at inhibiting phosphate-induced calcium deposition than alendronate and pamidronate, with an IC50 value (a quantitative measure of the concentration needed to inhibit calcification in vitro by 50%) of 1.16 μmol/L (47). Thus, etidronate would appear to be a better choice than other bisphosphonates, although the survival curves between etidronate versus other bisphosphonates do not appear to differ (28). Lastly, etidronate is no longer available in many countries.

Another therapeutic option to address the ectopic calcification is sodium thiosulfate, which increases the solubility of calcium. A combination of etidronate and sodium thiosulfate was given to an infant with GACI, with no improvement of calcification and demise after 1 month (48). The infusion of intravenous sodium thiosulfate led to improvement of calcific stenosis of celiac and mesenteric arteries in a child with a complex genotype (49). Anecdotally, several patients with ENPP1 deficiency have received intravenous sodium thiosulfate, but its benefits remain unclear given the lack of additional reports.

Increased dietary magnesium prenatally (given to pregnant dams) and continued postnatally was shown to prevent ectopic mineralization in a mouse model of ENPP1 deficiency, likely due to the competition of magnesium with calcium for phosphate binding (50). However, clinical trials testing the efficacy of magnesium supplementation on vascular calcification in PXE patients in a double-blinded placebo-controlled trial failed to show a statistically significant clinical benefit (ClinicalTrials.gov identifier NCT01525875) (51). One infant with ENPP1 deficiency who did not initially respond to etidronate administration eventually showed improvement in ectopic calcification (arterial and periarticular) with a combination of etidronate, magnesium, and calcium carbonate, with the latter as an antiphosphate treatment (52). However, it is possible that this infant could have shown spontaneous regression of calcification even in the absence of magnesium administration.

It should be noted that the therapeutic options mentioned above (bisphosphonates, sodium thiosulfate, and magnesium) address only the arterial calcification and not any other aspects of the cardiovascular involvement related to GACI. Standard of care for other potential complications such as cardiac failure, hypertension, and coronary artery stenosis is also warranted.

Periarticular calcification is seen in 64% of affected individuals, affecting in decreasing order the knee (27%), hip (27%), shoulder (27%), wrist (22%), sternoclavicular (16%), carpal (11%), elbow (9%), and ankle (9%) joints (28). These periarticular calcific masses can be large, sometimes leading to confusion with another rare disease of ectopic calcification, namely familial tumoral calcinosis. Calcifications around the joints can lead to pain and decreased range of motion of the affected joint. In some cases, these periarticular calcifications have remained stable for years (53). Calcification of the earlobes is seen in 55% of patients (54), while parenchymal calcification of organs is seen in 66% of patients (28). Therapeutic strategies for treating periarticular calcification include surgical debulking, which has been performed anecdotally for large calcific masses that do not regress over time and interfere with function.

## 3. PHOSPHATE WASTING RICKETS INDUCED BY ENPP1 DEFICIENCY: ARHR2

In 2010, ENPP1-deficient patients were found to develop fibroblast growth factor 23 (FGF23)-mediated phosphate wasting rickets (ARHR2) (11, 12). Phosphate wasting in ARHR2 patients is nearly identical to the phosphate wasting in the prototypic renal phosphate wasting disorder X-linked hypophosphatemia (XLH), due to pathogenic variants in *PHEX* (phosphate regulating endopeptidase homolog, X-linked). *PHEX* encodes an enzyme that degrades small integrin-binding ligand N-linked glycoproteins, or SIBLING proteins, but is thought to regulate *FGF23* expression through nonenzymatic mechanisms, and FGF23 regulates phosphate homeostasis by controlling the expression of sodium–phosphate cotransporters (NPT2a and NPT2c) on the apical membrane of proximal renal tubule cells. Elevated levels of FGF23 suppress the expression of NPT2a and NPT2c, decreasing phosphate resorption and thereby inducing phosphate wasting rickets. The finding of FGF23 phosphate wasting rickets in patients with inactivating ENPP1 variants led to speculation that *ENPP1* regulated *FGF23*, a hypothesis supported by RNA sequencing experiments demonstrating *FGF23* to be the single most elevated transcriptome in the bones of 10- and 23-week-old Enpp1-deficient mice (55).

**Figure 2:**
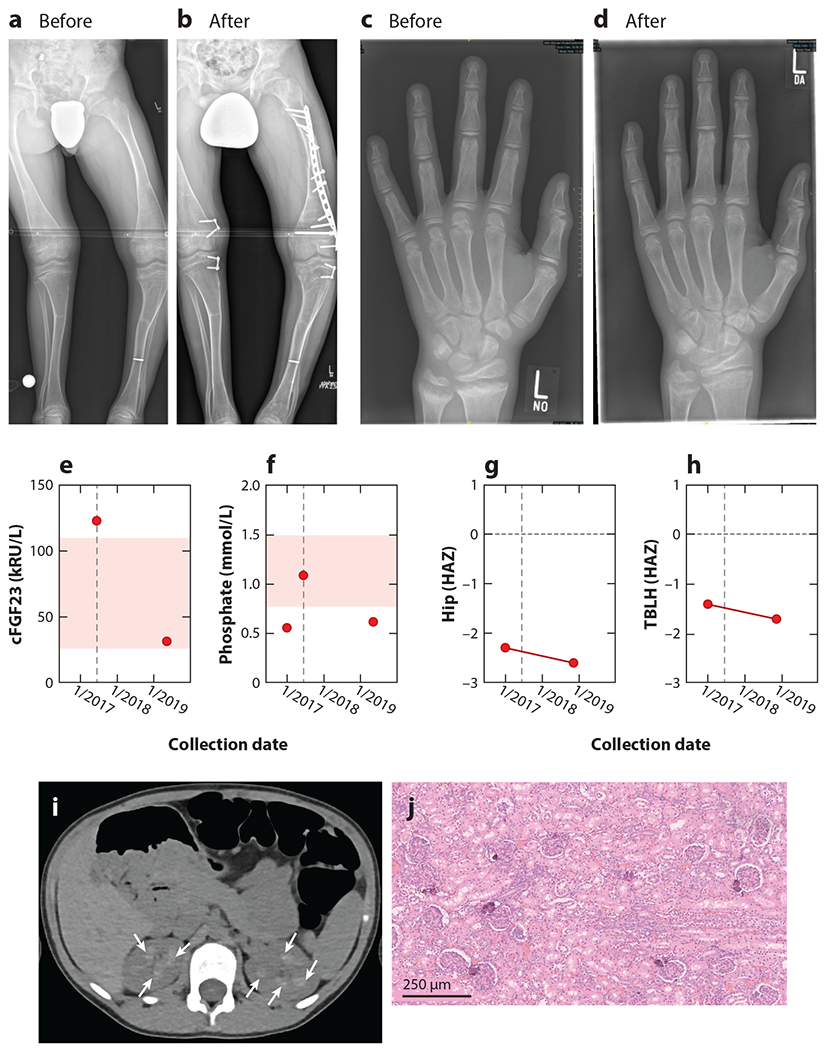
Skeletal phenotype of a 15-year-old autosomal recessive hyperphosphatemic rickets type 2 (ARHR2) patient before and after surgical correction and phosphate supplementation. (*a*) Prominent skeletal deformities with bending of long bones in lower limb were corrected by bilateral hemiepiphysiodesis, (*b*) requiring open reduction and internal fixation by a locking compression plate and traumatic diaphyseal femur fracture. (*c*) Radiograph of left hand and distal forearm of the patient at 14 years of age with fraying, spraying, and cupping of the metaphysis of the distal radius and ulna, which (*d*) was observed to improve following 2 years of supplemental phosphate and calcitriol therapy. The institution of supplemental phosphate and calcitriol (dashed line in panels *e* and *f*) reduced c-terminal fibroblast growth factor 23 (cFGF23) in plasma [measured in kilo–relative units per liter (kRU/L)] (*e*) and maintained or increased serum phosphate (*f*), but bone mass continued to decrease in the hip (*g*) and total body less head (TBLH) (*h*), as quantitated by the height-adjusted Z-score (HAZ). (*i*) Computed tomography of the abdomen of an ARHR2 child (aged 8 years, 3 months) treated with supplemental calcitriol and phosphate revealing bilateral calcification of the renal pyramids. (*j*) Renal histology of a deceased generalized arterial calcification of infancy (GACI) infant revealing foci of calcifications within the renal cortex. Figure adapted from Reference 57.

Children with XLH and ARHR2 typically present with very similar clinical phenotypes—bowing deformities of the legs, anteromedial rotational torsion of tibiae, and short stature (Figure 2a). Typical biochemical findings include hypophosphatemia and low-normal circulating 1,25-dihydroxyvitamin D [1,25(OH)_2_D]. Plasma levels of intact FGF23 are at the upper limits of normal or slightly elevated in the presence of low serum phosphate. The original reports of ARHR2 by Levy-Litan and colleagues (11) described three patients with rickets, two of whom had FGF23 levels at the upper limit of normal—50 pg/mL and 47 pg/mL (reference range of 10–50 pg/mL). A contemporaneous report by Lorenz-Depiereux (12) described seven patients, five of whom had intact plasma FGF23 at either the upper range of normal or slightly elevated upon repeated measurement and in any case inappropriate for the hypophosphatemia. All patients, however, exhibited clinical signs of rickets, as evidenced by the need to perform corrective surgery to address the bone deformities in three of the seven patients of Lorenz-Depiereux, while two of the three patients reported by Levy-Litan exhibited typical rachitic skeletal deformities. Finally, serum alkaline phosphatase is typically elevated, and renal tubular phosphate reabsorption is typically decreased, as defined by a low ratio of the tubular maximum threshold for phosphate reabsorption to glomerular filtration rate.

### 3.1. Clinical Presentation of ARHR2

The first biochemical manifestation of rickets is a decrease in serum phosphate. This is a progressive development—serum phosphate concentrations in affected patients at birth are normal. However, a sharp decline is observed after birth, with a mean rate of decline of −1.45 SD per year (95% CI = −1.90 to −1.00) that slows over time (mean change in rate = 0.12 SD per year, 95% CI = 0.06 to 0.18) (16). The average onset of hypophosphatemia (serum phosphate Z-score <−1.96) is 1.6 years of age. Intact FGF23 concentrations are frankly elevated (>50 pg/mL) in most patients (88%) and correlate significantly and inversely with serum phosphate. In addition, patients with elevated intact FGF23 concentrations have inappropriately suppressed levels of 1,25(OH)_2_D (27).

Clinical signs of rickets are seen in 49% of individuals with ENPP1 deficiency regardless of age or survival status and in 70.1% of patients who survive the critical period (28). It should be noted that the risk of developing rickets varies with age, with an estimated probability of developing rickets of 20% by 2 years of age and an almost certainty of developing rickets by adolescence or osteomalacia in early adulthood (27). Rickets not only is seen in survivors of GACI but can also be diagnosed as the first manifestation of ENPP1 deficiency, in the absence of any known prior cardiovascular involvement; however, 64% of individuals in this group had evidence of ectopic calcification (arterial, valvular, or periarticular) after further studies were obtained. The median age for the diagnosis of rickets in this subcohort is 5.4 years (28).

### 3.2. ARHR2 Therapeutic Strategies

Treatment of hypophosphatemic rickets is often accomplished with calcitriol plus frequent administration of phosphate supplements, which corrects the rickets phenotype (compare Figure 2c, before treatment, with Figure 2d, after treatment), but judicious treatment is necessary to avoid the development of hyperparathyroidism and hypercalciuria without worsening vascular calcification (27, 56). However, nephrocalcinosis is a known complication of this treatment strategy (Figure 2i). In one study, medullary nephrocalcinosis was not present by ultrasound in any patient naive to treatment, while it developed in some subjects after initiation of treatment (*p* = 0.04, Student’s two-tailed t test). The presence of nephrocalcinosis was not related to age (which was statistically similar in patients with and without nephrocalcinosis), thus confirming an association of standard therapy with nephrocalcinosis (57). Anecdotally, the medullary nephrocalcinosis has not progressed to either glomerular or tubular renal dysfunction.

Burosumab is a monoclonal antibody against FGF23 that was developed to treat XLH patients. Since rickets related to ENPP1 deficiency is also mediated by FGF23, the use of burosumab has been proposed as a therapeutic strategy in ARHR2 patients. However, FGF23 is associated with suppression of alkaline phosphatase activity (58), so that FGF23 inhibition might lead to an increase in alkaline phosphatase activity, which may result in further decreases in PP_i_ levels.

More concerning, however, is the possibility that FGF23 elevations in ENPP1 deficiency are an adaptive, rather than pathologic, physiologic response to low plasma PP_i_ levels. This scenario suggests that a physiologic set point in the P_i_/PP_i_ ratio is able to balance mineralization activators and inhibitors. Thus, the organisms would be inclined to waste phosphate when plasma PP_i_ is reduced, thereby preventing vascular calcification at the expense of skeletal mineralization. If such a physiologic set point exists, we would expect burosumab to exacerbate vascular calcifications in ARHR2 patients, and indeed this was the case for one ARHR2 patient treated with burosumab. Echocardiographic studies revealed that this patient manifested two stable calcified nodes on the aortic valve without evidence of aortic stenosis or regurgitation, and computed tomography demonstrated calcifications of the aortic valve leaflets and mitral annulus with no calcifications of the coronary arteries. Four months after initiation of burosumab therapy, echocardiographic findings were stable, but 20 months after onset of therapy, significant calcification of the right and noncoronary cusps of the aortic valve with mild aortic stenosis, extensive calcification of the left ventricular outflow tract with a 5 × 6-mm calcified nodule, and calcification of the posterior septum, inferior wall, and posterior medial papillary muscle of the left ventricle were evident (59). It should be noted that serum alkaline phosphatase activity was not elevated after the use of burosumab in this patient, but systemic alkaline phosphatase activity may not reflect the local microenvironment of the surrounding cells. Indeed, local upregulation of alkaline phosphatase in vessels has been shown to lead to vascular calcification (60, 61), whereas transgenic mice with a >10-fold increase in serum alkaline phosphatase activity had no histological evidence of ectopic mineralization (62). Regardless of whether physiologic mechanisms balancing the P_i_/PP_i_ ratio in humans are present, it seems prudent to avoid burosumab in patients with ARHR2 because of concerns that it may lead to worsening ectopic calcification.

## 4. MUSCULOSKELETAL COMPLICATIONS INDUCED BY ENPP1 DEFICIENCY

### 4.1. Enthesis and Enthesopathies

Fibrocartilaginous entheses (tendon and ligament insertion sites) have evolved to accommodate mechanical loads (63). Thus, enthesis fibrocartilage is adapted to accommodate the distribution of forces between tendon/bone interfaces and is characterized by considerable tensile and compressive strength. Fibrocartilage is suited to function in this capacity: The zone of unmineralized fibrocartilage is rich in type II collagen, allowing for flexibility, whereas the zone of mineralized fibrocartilage composed of type II and X collagens, as well as proteoglycans, imparts compressive strength (64, 65). Thus, the extracellular matrix composition of the structure is related to its unique function.

Importantly, patients with ARHR2 and XLH exhibit progressive calcifications of entheses and form enthesophytes, or bony spurs, at insertion sites (66), which can be extremely painful and limit joint range of motion. Related overlapping features of XLH and ARHR2 include radiographic evidence of articular cartilage degeneration, subchondral sclerosis, pervasive osteophyte formation, and other features of degenerative osteoarthritis (67, 68). Both enthesopathy and osteoarthritis in XLH and ARHR increase with age and cause severe pain, stiffness, and difficulty performing tasks of everyday life. Enthesis-related complications are therefore of considerable clinical importance and represent significant morbidity in these patients (67–73). These occur frequently, particularly at the knees, ankles, pelvis, and thoracic/cervical spine sites, typically increasing with age (70).

Moreover, calcifications may occur in the spinal ligaments, with associated spinal stenosis (67, 70, 74). There is a strong relationship between formation of osteophytes, lateral exostoses at the synovial joint, and articular cartilage degeneration (75, 76). Similarly, patients with ARHR2 exhibit osteoarthritis, interosseous ossification, enthesopathies, and spinal fusion (73). Cervical fusion may occur in ARHR2, affecting approximately a quarter of patients and mainly involving the posterior vertebral elements including the posterior vertebral bodies, articular processes, and laminae (27) consistent with changes seen in XLH. In both XLH and ARHR2 these changes are not evident in infancy, but they may develop over the life span of affected patients.

Residual pain from ARHR2-related enthesopathies, as measured by the Brief Pain Inventory–Short Form, is similar in magnitude to that identified in adult patients with XLH and is experienced by the majority of ARHR2 patients despite use of analgesic medications. This pain is associated with impairment of physical function when using a Patient Reported Outcome Measurement Information System^®^ Physical Function (PROMIS PF) short form; this impairment in physical function varied from mild to severe, with the majority of patients having moderate impairment (73). Despite ample radiographic evidence of these frequent and severe complications, there is little understanding of the accompanying cellular pathophysiology. Moreover, therapies have been directed toward childhood management, with little emphasis on adult complications.

### 4.2. The Role of FGF23 in Enthesopathy

Increased FGF23 is present in rare diseases inducing enthesopathy, such as XLH and ARHR (11, 12, 72, 73), findings that implicate a potential causative role for FGF23 or the resultant hypophosphatemia in its pathogenesis. However, the means by which elevated FGF23 and/or reduced phosphate may induce enthesopathy is not clear.

Excessive enthesis mineralization has also been described in other FGF23-dependent hypophosphatemic disorders, including ARHR1 and tumor-induced osteomalacia (77). Of interest, in OPLL, elevated FGF23 and low plasma P_i_ can occur in addition to enthesis mineralization (78, 79). Indeed, transgenic mice overexpressing the secreted form of human FGF23 exhibit enthesopathy (72), and entheseal fibrochondrocytes are known to express the FGFR3 receptor as well as the Klotho coreceptor, which promotes FGF23 signaling (70). FGF23 thus appears to be intimately related to the development of certain forms of heterotopic mineralization, although it is not clear whether this effect is directly or indirectly mediated. In contrast, enthesopathy is not described in FGF23-independent forms of hypophosphatemic rickets, such as hereditary hypophosphatemic rickets with hypercalciuria due to pathogenic variants in *SLC34A3*, which encodes the renal tubular NPT2 phosphate transporter (63). One hypothesis is that FGF23-mediated suppression of 1,25(OH)_2_D synthesis leads to enhanced bone morphogenic protein and Indian hedgehog signaling, with subsequent enthesis calcification (64). Other possibilities such as the direct suppression of ENPP1 by FGF23 resulting in reduced PP_i_ have also been suggested (55).

### 4.3. Spinal Hyperostosis in ENPP1 Deficiency

OPLL and DISH are relatively common age-related musculoskeletal diseases inducing pain, reduced range of motion, spinal fractures, and, in severe cases, hemiplegia (80–82). DISH becomes prevalent after age 50 and affects more than a quarter of men and women after age 80, while OPLL is prevalent in Americans with cervical myelopathy and even more extensively in the Asian population. Myelopathy and decreased mobility progressively worsen with age, and treatment usually consists of conservative chronic pain management with NSAIDs as there are no effective therapies preventing the progressive ossification responsible for the symptomatic course (83). Despite the identification of Enpp1 deficiency in murine models with an OPLL-like phenotype (32), only a single ENPP1 polymorphism has been associated with OPLL in humans to date (84). Moreover, large-scale genome-wide association studies in humans identified six susceptibility OPLL loci, none of which contain *ENPP1* (85), and sibling pair linkage analysis failed to establish an association between OPLL and *ENPP1* (86). Although increased FGF23 correlates with OPLL progression, causation has not been established. Of note, however, spinal enthesopathies and spinal fusion occur in patients with OPLL and DISH in a manner reminiscent of those with ARHR2 and XLH (80, 87, 88), suggesting that the disorders may be related.

**Figure 3:**
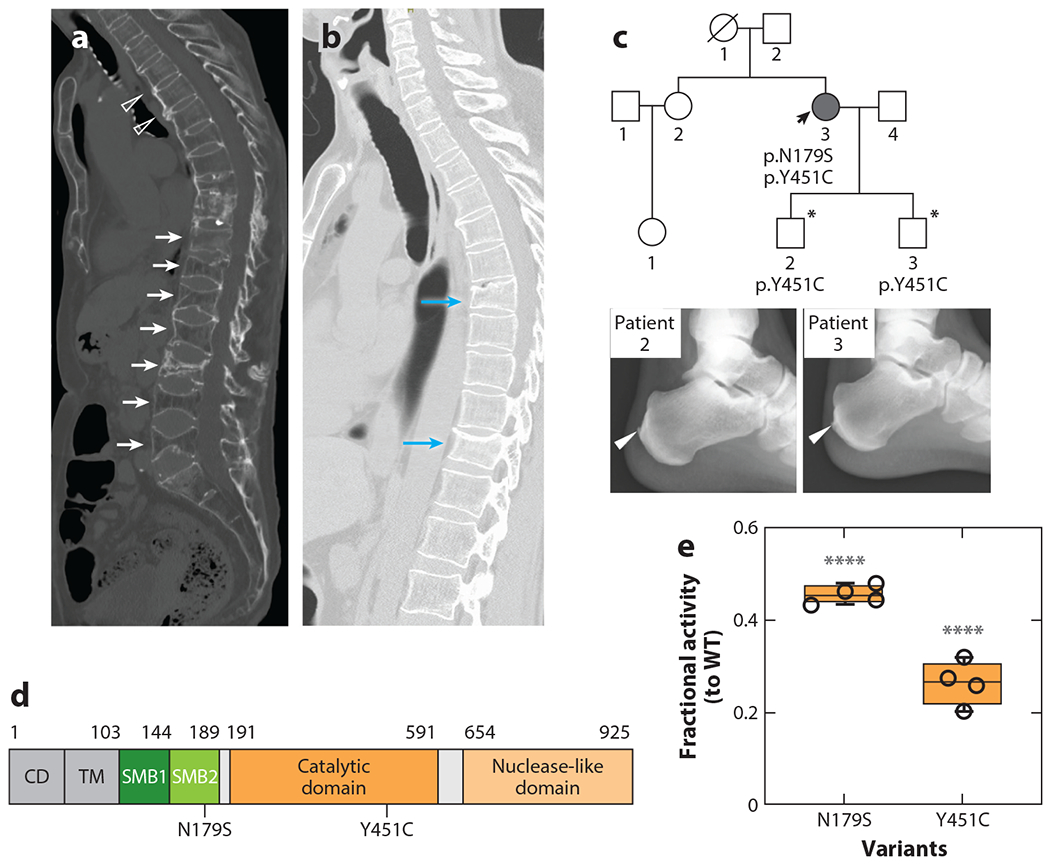
Spinal and Achilles enthesopathies in diffuse idiopathic skeletal hyperostosis patients with ENPP1 haploinsufficiency. (*a*) Multiple paraspinal ligament ossifications (*open arrowheads*) and compression fractures (*white arrows*) in a patient with a heterozygous ENPP1 Y451C pathogenic variant. (*b*) Multiple compression fractures in the spine of a second patient with a heterozygous ENPP1 N179S pathogenic variant (*blue arrows*). (*c*) Pedigree of a patient (*black arrow*) with compound *ENPP1* pathogenic variants, one of which (Y451C) was passed on to both sons (*asterisks*), who both exhibited Achilles tendon enthesis calcification at the ages of 19 and 23 years. (*d*) Schematic of ENPP1 illustrating the location of variants (in SMB2 and the catalytic domain). The nuclease domain is omitted to conserve space. (*e*) When compared with WT ENPP1, the N179S and Y451C variants reduced activity by 55% and 70%, respectively (**** indicates *p* < 0.0001). Abbreviations: CD, cytoplasmic domain; ENPP1, ectonucleotide pyrophosphatase/phosphodiesterase 1; SMB, somatomedin B-like domain; TM, transmembrane domain; WT, wild type. Figure adapted from Reference 89.

To investigate the possibility that ENPP1 deficiency may be an unrecognized contributing factor to spinal enthesopathies in DISH and OPLL, a clinical study examining the skeletal, biochemical, and genetic findings in patients with DISH was performed to investigate the relationship between FGF23, plasma PP_i_, spinal disease, and genetic background (89). All patients had been previously diagnosed with DISH and presented with pain, progressive immobility, osteoarthritis, and/or compression fractures of the spine, and all manifested heterotopic calcifications in their paraspinal ligaments, Achilles tendons, and joints. The plasma biochemistries were characterized by low-normal phosphate and high-normal FGF23, which invoked the association of elevated FGF23 and rapidly progressing OPLL (78, 79) as well as the plasma biochemical profile of patients with ENPP1 haploinsufficiency (90). Genetic sequencing using a panel of genes associated with bone/mineralization disorders found compound heterozygous variants in *ENPP1* in one patient (c.536A>G and c.1352A>G; p. N179S and p. Y451C), and missense *ENPP1* variants in two additional patients resulting in ENPP1 Y451C and N179S heterozygous individuals (Figure 3a,b).

Biochemical assays revealed that the N179S and Y451C ENPP1 variants reduced enzyme velocity by 45% and 30%, respectively, suggesting that decreased PP_i_ could account for the observed spinal enthesopathies (Figure 3e). Sanger sequencing for the variants identified in the first patient was then performed in her two sons (19 years old and 23 years old), both of whom were found to possess heterozygous Y451C ENPP1 pathogenic variants and Achilles tendon enthesopathies at their young age (Figure 3c), further demonstrating the association of enthesopathy and ENPP1 pathogenic variants.

In summary, this small pilot study demonstrated the surprising prevalence of ENPP1 deficiency in patients carrying the diagnosis of DISH. Moreover, the allele frequency of the Y451C ENPP1 variant is approximately 30 times higher in the Japanese population (ToMMo: 0.0055) than in all other race/ethnicity groups (GnomAD: 0.00016). These observations suggest that different frequencies for occurrence of the Y451C variant could explain the 10–40 times higher prevalence of OPLL in the Japanese population (1.8–6.4%) as compared with the US (0.12%) and German (0.1%) populations (91, 92), observations suggesting that ENPP1 pathogenic variants may be driving the enthesopathies in a subset of DISH and OPLL patients.

Current therapies for the spinal enthesopathies present in XLH, ARHR2, OPLL, and DISH have no apparent effect on the initiation and progression of enthesopathy and osteoarthritis, and there is very little understanding of factors responsible for initiating and promoting the heterotopic mineralization. Decompression laminectomy is used to provide acute relief, but progression of entheses in OPLL occurs more rapidly after such surgery than in conservatively managed patients (70% versus 24%, respectively), discouraging surgical intervention in all but severely symptomatic cases (83, 93–96).

## 5. BONE MASS IN ENPP1 DEFICIENCY

**Figure 4:**
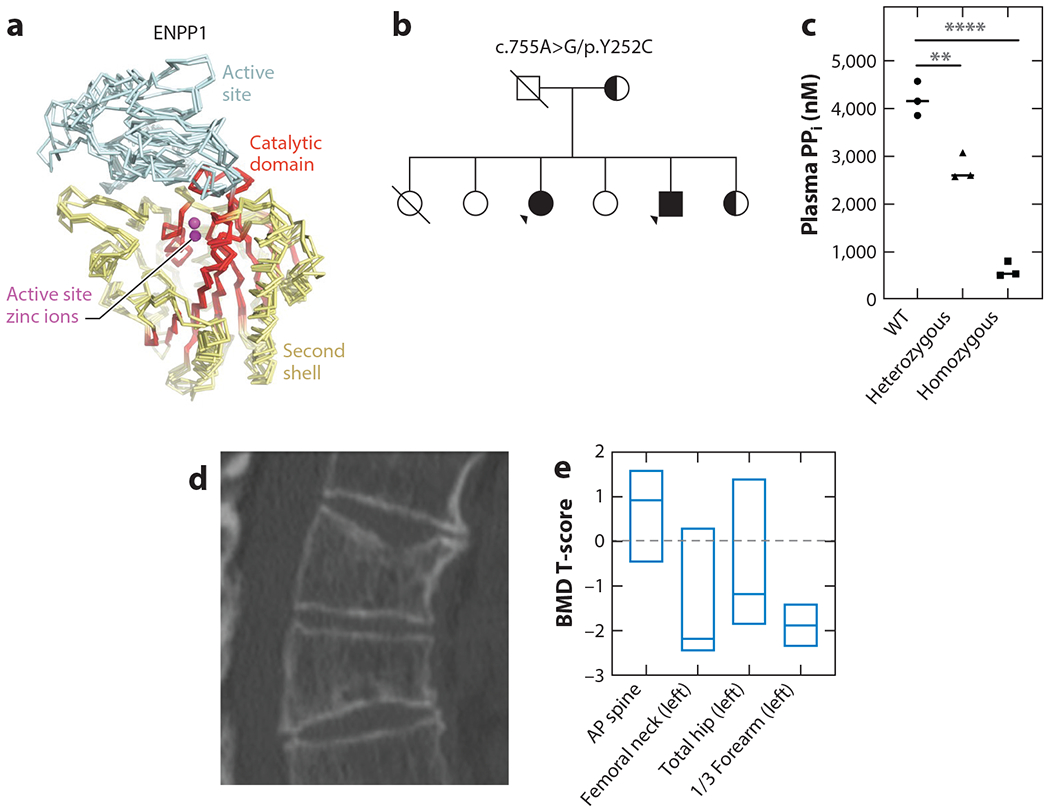
ENPP1 deficiency and low mammalian bone mass. (*a*) Superposition of Cα traces of human ENPP4 (PDB code 4LQY) with mouse ENPP1 (PDB code 4B56), mouse ENPP2 (PDB code 3NKN), and a bacterial ENPP (PDB code 2GSU) to illustrate the degree of structural conservation of the catalytic domain (with an average RMSD of 0.68 Å), which includes the active site zinc ions, the α-helix on which the catalytic threonine is located, and the backbone near the hydrophobic slot. Regions of moderate similarity include the second shell of the active site (average RMSD of 1.33 Å), and the lowest similarity is the substrate binding domain of the active site (average RMSD of 2.51 Å). The ccp4 program Superpose was used to overlay the conserved subdomain (124). Active site zinc ions are depicted as spheres. (*b*) Pedigree of a family possessing individuals with homozygous and heterozygous ENPP1 Y451C pathogenic variants. (*c*) Plasma PP_i_ levels of the family members plotted by their mutational status. (*d*) Micro-CT image of thoracic spine in a patient with an ENPP1 heterozygous Y451C pathogenic variant revealing multiple compression fractures. (*e*) Areal bone mineral density T-scores in a patient with ARHR2, displayed as box plots denoting median value and interquartile range. Abbreviations: AP, anteroposterior; ARHR2, autosomal recessive hyperphosphatemic rickets type 2; BMD, bone mineral density; micro-CT, microcomputed tomography; ENPP1, ectonucleotide pyrophosphatase/phosphodiesterase 1; PDB, Protein Data Bank; PP_i_, pyrophosphate; RMSD, root mean square deviation; WT, wild type. Figure adapted from References 37 and 90.

Although low bone mass has been described in several murine models of Enpp1 deficiency, including *ttw* mice (33), *ENPP1* knockout (KO) mice (*Enpp1*^−/−^) (97), and an *ENPP1* C397S mutant mouse discovered as part of a large genome-wide mutagenesis screen to identify genes regulating bone mass (98), osteoporosis in ENPP1-deficient patients had not been noted until recently. Thus, the murine findings were discordant with the rachitic skeletal phenotype described in humans with biallelic *ENPP1* pathogenic variants. However, this changed in 2019 when Ralf Oheim at the University of Hamburg–Eppendorf Medical Center identified three patients with early-onset osteoporosis with heterozygous *ENPP1* pathogenic variants (90). These patients had either Y471C or H777C *ENPP1* variants, both of which had been observed in GACI patients. Two of the three patients presented with multiple vertebral fractures (Figure 4d) and hip and/or spine bone mineral density (BMD) in the osteoporotic range. All three patients where hypophosphatemic and exhibited inappropriately high or high-normal circulating FGF23 levels (two of the three patients had frankly elevated FGF23 levels). Thus, these osteoporotic individuals with biallelic ENPP1 haploinsufficiency exhibited phosphate wasting typical of that observed in ARHR2 patients with biallelic ENPP1 deficiency. Finally, plasma PP_i_ as measured in family members without and with *ENPP1* heterozygous and homozygous pathogenic variants showed greater values in individuals without *ENPP1* pathogenic variants than in individuals with heterozygous variants, who in turn exhibited greater PP_i_ levels than those with homozygous variants (Figure 4c). The Y471C and H777C variant alleles were found to reduce ENPP1 enzymatic velocity by 70% and 95% compared with the WT enzyme, respectively, suggesting that ENPP1 deficiency may induce gene-dose effects, such that the variable degrees of ENPP1 pathogenic variants can modify the phenotype: Phosphate wasting occurred in both biallelic and monoallelic deficient family members, but monoallelic members exhibited osteoporosis in middle age rather than rickets in infancy and adolescence. Similarly, observations in another extended family carrying two ENPP1 variants exhibited low to low-normal plasma phosphorous in siblings with monoallelic ENPP1 pathogenic variants—C108F and R481W—which reduced enzymatic velocity by 80% and 45%, respectively. Although bone mass was not measured, reduced plasma pyrophosphate was also found in both monoallelic patients, further supporting an *ENPP1* gene-dose effect. Moreover, low bone mass was also seen in the adults in the National Institutes of Health cohort with biallelic ENPP1 deficiency, who all met criteria for osteopenia or osteoporosis in at least one site—two patients were osteopenic (T-score between −1.0 and −2.5) in the distal radius and total hip, and one patient each had osteopenia and osteoporosis (T-score of −2.5 or lower) at the femoral neck (57) (Figure 4e).

**Figure 5:**
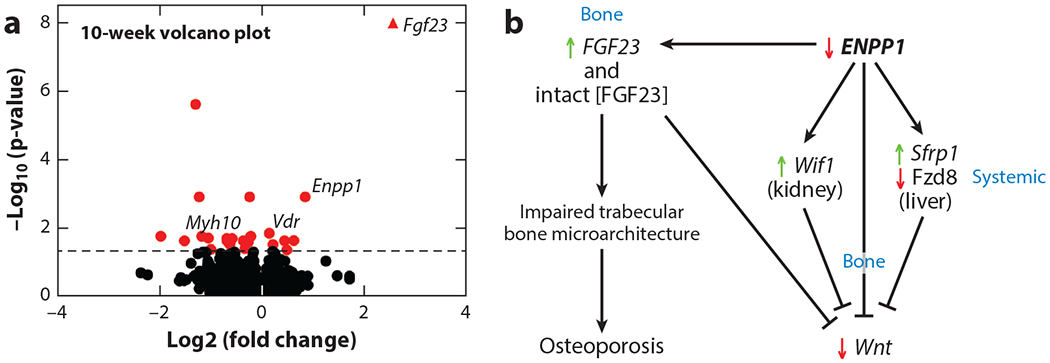
Differential gene expression in the tibias of 10-week-old male *Enpp1*^*asj*^ and WT mice analyzed by RNA sequencing, (*a*) Volcano plots to illustrate the differential gene expression in 10-week-old male *Enpp1*^*asj*^ mice and WT siblings reveal the most upregulated transcript to be *FGF23*. (*b*) Transcriptome analysis of bone mineralization pathways disrupted by murine Enpp1 deficiency reveals suppression of *Wnt* due to increased expression of the *Wnt* inhibitors *Wif1* in kidney and *Sfrp1* in liver and decreased transcription of *Wnt* ligand *Fzd8* in liver. Increased FGF23 in bone further inhibits bone mineralization through inhibition of trabecular bone (125) and suppression of *Wnt* (126). Abbreviations: ENPP1, ectonucleotide pyrophosphatase/phosphodiesterase 1; FGF23, fibroblast growth factor 23; WT, wild type. Figure adapted from Reference 55.

Evidence of an FGF23-mediated phosphate wasting disorder and osteoporosis also is observed in *Enpp1^asj^* mice, which also exhibit increased plasma intact FGF23, low plasma phosphate, and osteoporosis at 10 and 23 weeks of age on a regular chow diet. Transcriptome analysis of the bones of these mice at 10 and 23 weeks revealed reduced gene transcription of Wnt ligands in whole bones (Wnt16b and Wnt10b) as well as increased transcription of soluble Wnt inhibitors in the liver (*Sfrp1*) and kidney (*Wif1*), suggesting multiorgan Wnt inhibition (Table 2) at both time points. Consistent with Wnt suppression, collagen gene pathways in bone were also significantly reduced and Fgf23 was significantly elevated (Figure 5), findings that correlated with pronounced reductions in trabecular bone and increased fracture risk in the *Enpp1*^*asj*^ mice (55, 90).

Although osteoporosis in patients with heterozygous ENPP1 pathogenic variants demonstrates a critical role for the enzyme in normal skeletal development, the mechanism by which ENPP1 regulates bone mass is unknown. Various mechanisms for the low bone mass observed in murine models have been suggested. These include claims that PP_i_ may serve as a substrate for rapid conversion to P_i_ (99). Others suggest that PP_i_ deficiency depletes the mineralization inhibitor in the axial skeleton, resulting in enhanced calcification of the osteocyte lacunae and canaliculi, which restricts blood flow and compromises osteocyte mechanosensing (100). Finally, an early in vitro study found that ENPP1 protein, but not extracellular PP_i_, is required for osteoblastic differentiation, raising considerations that catalysis-independent ENPP1 signaling may regulate osteoblast maturation and differentiation, with a subsequent impact on bone mass (101). To this point, ENPP1, in addition to its catalytic domain, possesses somatomedin domains that mediate protein interactions.

**Figure 6:**
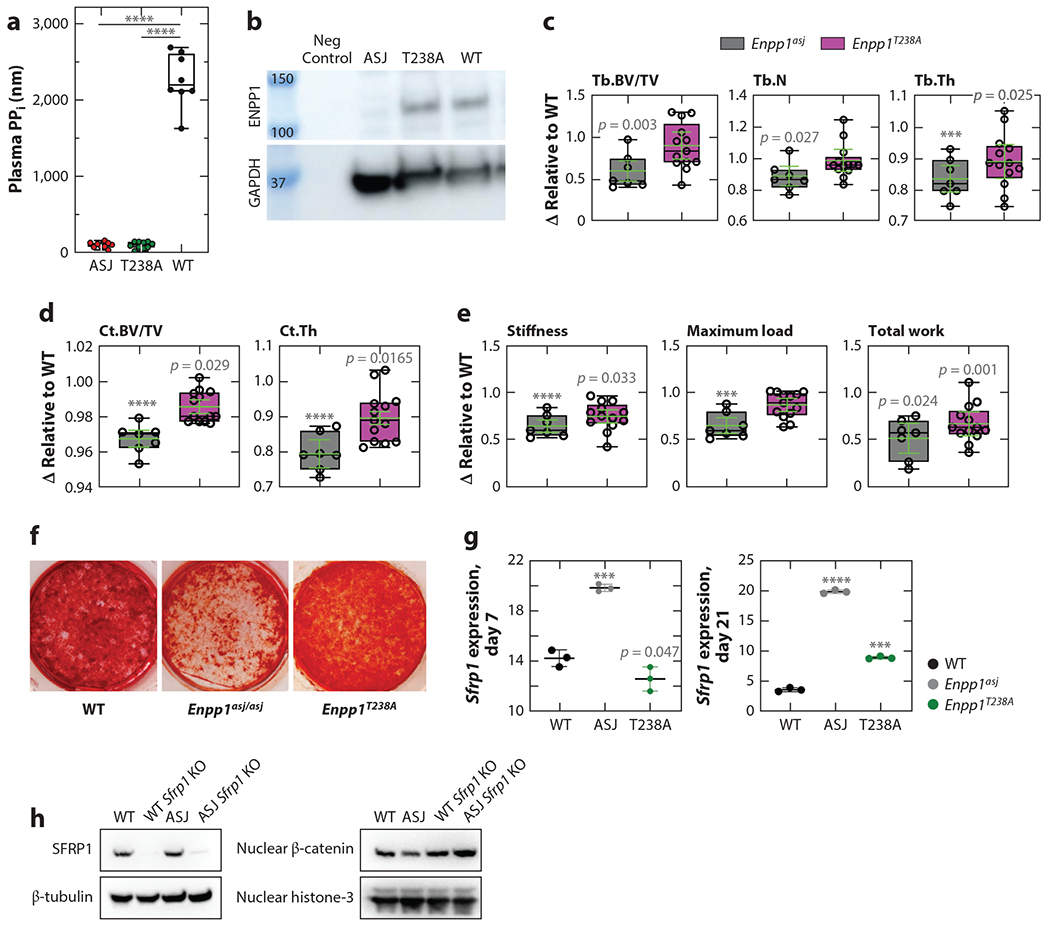
The T238A mouse. (*a*) Plasma PP_i_ was absent in both *Enpp1*^*T238A*^ and *Enpp1*^*asj*^ mice, demonstrating abrogation of ENPP1 catalytic activity in both genotypes. (*b*) Western blot of primary osteocytes derived from calvaria cells confirms comparable ENPP1 protein expression in T238A and WT mice and reduced expression in ASJ mice (normalized to GAPDH). Analysis of skeletal mineralization demonstrated preservation of trabecular (*c*), and cortical (*d*) mineralization parameters as well as biomechanical performance (*e*) in the tibias and femurs of *Enpp1*^*T238A*^ mice, as compared with *Enpp1*^*asj*^ mice. The data in panels *c–e* are displayed as change (Δ) relative to WT siblings using box plots to display median values and interquartile range. Superimposed on the box plots in green is the relative mean (*bar*) and standard error of the mean (*whiskers*) for each measurement. (*f*) Calvaria cells derived from WT, *Enpp1*^*asj*^, and *Enpp1*^*T238A*^ mice and differentiated into osteocytes in a phosphate source for 21 days exhibit dramatic reductions in calcium phosphate in the *Enpp1*^*asj*^ mice (stain, Alizarin Red). (*g*) Quantitation of mRNA expression of *Sfrp1* in calvaria osteoblasts derived from WT mice (*black symbols*) and *Enpp1*^*asj*^ mice (*gray symbols*) reveals elevations of Sfrp1 at days 7 and 21. Expression normalized to Hprt1. (*h*) Western blot of *Sfrp1* knockout in *Enpp1*^*asj*^ calvaria cells demonstrates increased nuclear β-catenin, demonstrating that suppression of β-catenin signaling via SFRP1 accounts for the mineralization defect in ENPP1 deficiency. Values of *p* are explicitly stated as 0.05 ≥ *p* ≥ 0.001; *** indicates *p* < 0.001 and **** indicates *p* < 0.0001, Student’s unpaired t test (to respective WT sibling pairs). Abbreviations: ASJ, associated with stiff joints; BV/TV, bone volume to total volume fraction; Ct.Th, cortical thickness; ENPP1, ectonucleotide pyrophosphatase/phosphodiesterase 1; GAPDH, glyceraldehyde 3-phosphate dehydrogenase; PP_i_, pyrophosphate; Tb.N, trabecular number; Tb.Th, trabecular thickness; WT, wild type. Figure adapted from Reference 102.

To investigate the presence of catalysis-independent pathways, a conservative point variant was introduced in the catalytic residue of murine Enpp1 to generate *Enpp1*^*T238A*^ transgenic mice (so named for the substitution of an alanine for threonine at residue 238). Plasma PP_i_ levels in *Enpp1*^*T238A*^ mice are approximately 5% of WT levels, but osteoblast ENPP1 protein expression is comparable to WT siblings (Figure 6a), and therefore this variant eliminated the catalytic activity but preserved protein expression, thus allowing for the retention of catalysis-independent protein signaling (102). The skeletal phenotype in *Enpp1*^*T238A*^ mice demonstrated normalization of trabecular and cortical microarchitecture in comparison with *Enpp1*^*asj*^ mice, which lack both catalytic activity and protein expression (Figure 6c,d). Moreover, biomechanical testing revealed that maximum load, bone stiffness, and total work until fracture were preserved in the femurs of 10-week-old *Enpp1^T238A^* mice compared with *Enpp1^asj^* mice (Figure 6e). The combined findings support the notion that catalytically independent ENPP1 pathways significantly contribute to mammalian bone mass.

**Figure 7:**
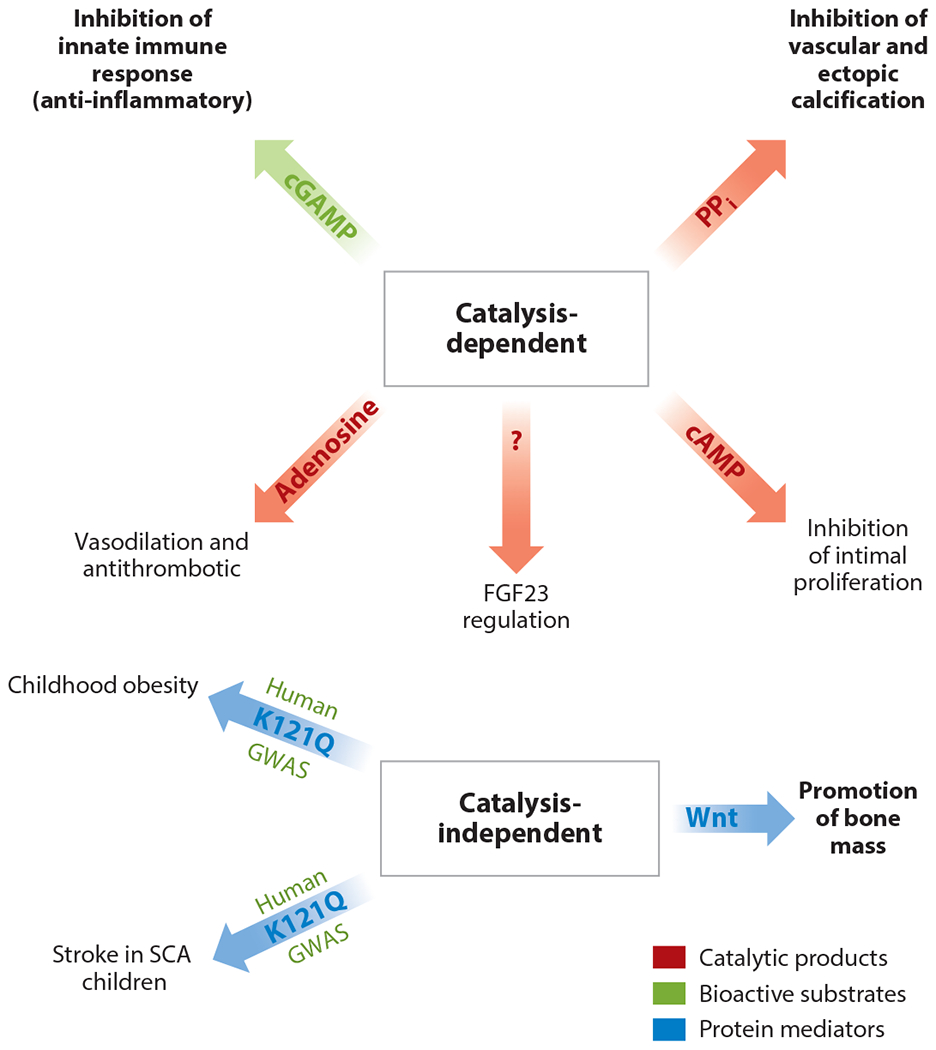
ENPP1 regulates organismal physiology though catalysis-dependent and -independent mechanisms. ENPP1 catalytic activity regulates whole organismal physiology by generating catalysis-dependent products (in *red*) or hydrolyzing bioactive substrates (in *green*). Catalysis-independent effects of ENPP1 are mediated through ENPP1 protein signaling and are related to a commonly occurring polymorphism in the ENPP1 somatomedin B-like domain 2 (SMB2) domain—K121Q—identified by GWAS to be associated with childhood obesity (127) and increase risk of stroke in SCA (128). Finally, ENPP1 catalysis-independent pathways regulating bone mass have been recently confirmed in animal models (102). Red type is used to label catalytic products, green type is used to label catalytic substrates of ENPP1 regulating the listed phenotypes, and blue type is used to label protein mediators regulating the listed phenotypes in the figure. Abbreviations: cAMP, cyclic adenosine monophosphate; cGAMP, cyclic guanosine monophosphate–adenosine monophosphate; ENPP1, ectonucleotide pyrophosphatase/phosphodiesterase 1; FGF23, fibroblast growth factor 23; GWAS, genome-wide association studies; PP_i_, pyrophosphate; SCA, sickle cell anemia.

Mechanisms regulating bone mass were investigated using mineralizing cell cultures derived from *Enpp1^T238A^* and *Enpp1^asj^* calvaria cell cultures. Calvaria cell cultures of *Enpp1^asj^* mice demonstrated markedly decreased calcification compared with *Enpp1^T238A^* mice, which calcified at or above WT levels (Figure 6f). Further studies identified a marked elevation of the Wnt inhibitor Sfrp1 (Figure 6g), although other Wnt inhibitors such as Dkk1 were also upregulated, and the expression of some Wnt ligands and receptors such as Wnt5, Wnt9, and Lrp5 was decreased. Wnt signaling was confirmed to be reduced by demonstrating reduced nuclear β-catenin in *Enpp1^asj^* osteoblasts by knocking out *Sfrp1*, which restored both mineralization in the differentiating cell cultures and the nuclear β-catenin signaling, supporting the notion that Sfrp1-mediated inhibition of *Wnt* is responsible for the osteopenia observed in ENPP1 deficiency (Figure 6h). Indeed, *Sfrp1* KO mice exhibit increased trabecular bone mineralization, further suggesting that elevated Sfrp1 in ENPP1 deficiency may be responsible for the trabecular mineralization defects observed (103). In summary, studies in *Enpp1^T238A^* mice demonstrated that catalysis-independent ENPP1 signaling pathways regulated mammalian bone mass through suppression of Wnt, which was seen to primarily occur through the elevation of Sfrp1. The catalysis-dependent and catalysis-independent effects of ENPP1 are summarized in Figure 7.

## 6. PROTEIN REPLACEMENT THERAPY

The constellation of clinical symptoms induced by ENPP1 deficiency is diverse, is life threatening, and involves seemingly opposing mineralization pathways that induce progressively increasing calcifications in the vasculature, tissues, and tendons, while simultaneously suppressing skeletal mineralization. The diverse clinical phenotypes induced by ENPP1 deficiency—vascular calcifications, vascular intimal endothelial cell hyperplasia, FGF23 mediated phosphate wasting rickets, osteoporosis, enthesis calcifications, spinal fusion, and hearing loss—are unlikely to be addressed by a single therapeutic intervention, or even a combination of interventions. Perhaps for these reasons, the current therapeutic interventions for ENPP1-deficient patients appear at best ineffective and at worst counterproductive. For example, the calcitriol and phosphate supplementation used to address rickets in ARHR2 does indeed improve the rickets but does not improve the low bone mass, and it places ARHR2 patients at risk for nephrolithiasis (57) (Figure 2e–i). Bisphosphonate therapy, which constitutes the mainstay of GACI treatment to combat life-threatening vascular calcifications in neonates, is without significant benefit unless administered within the first week of life (27) and has been associated with significant skeletal toxicity in growing infants (104). Proposed dietary interventions such as pyrophosphate and magnesium supplementation, while less harmful, are unlikely to yield a clinical response due to the limited half-life of these oral agents which, if they are to be clinically effective, must continuously inhibit the nucleation and propagation of a growing crystalline lattice to oppose the physical-chemical forces driving hydroxyapatite mineralization in the extracellular environment.

**Figure 8:**
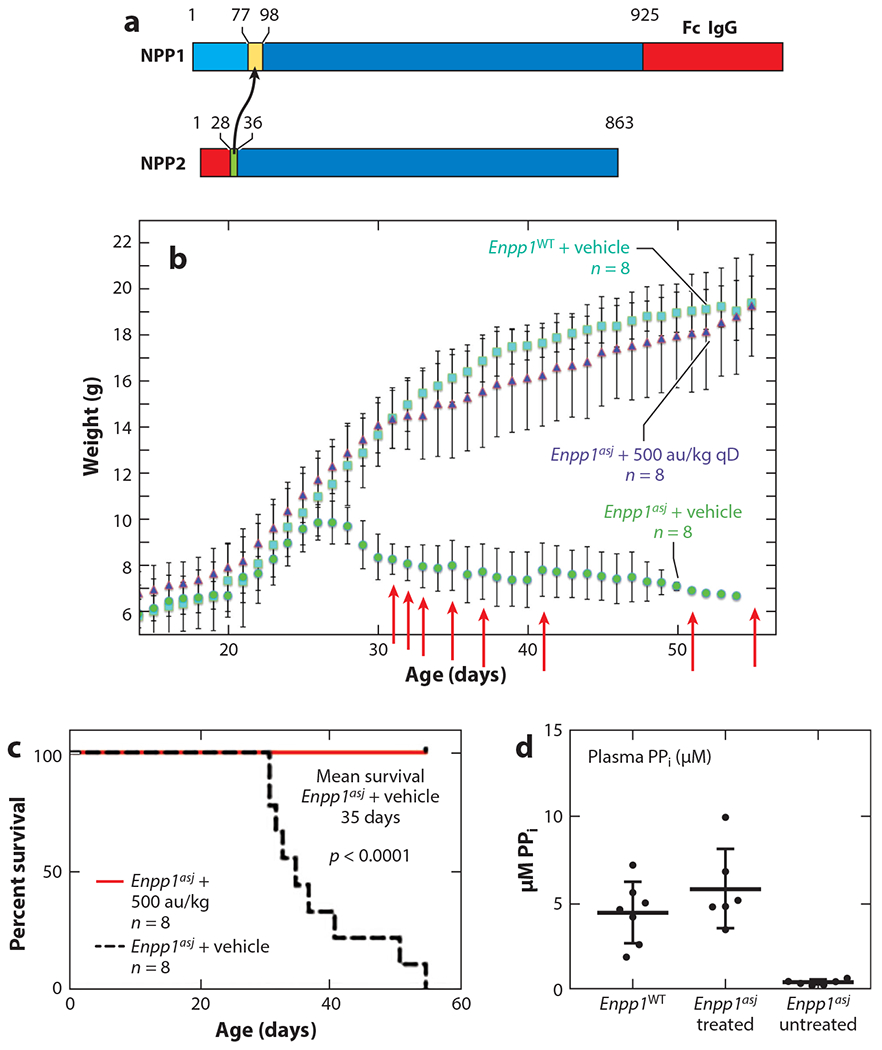
The efficacy of ectonucleotide pyrophosphatase/phosphodiesterase 1 (ENPP1) enzyme replacement in a murine model of generalized arterial calcification of infancy: biologic design, survival, and biomarkers. (*a*) Design of the first-generation murine Enpp1-Fc therapeutic. To produce a soluble recombinant protein, a segment of the extracellular region of NPP2 containing a furin cleavage site was substituted into ENPP1, and the Fc portion of immunoglobulin G1 (IgG1) was appended to the C-terminus. (*b*) The mean daily weights and standard deviations of wild-type (WT) *Enpp1*^WT^ (*squares, n* = 8), Enpp1-Fc-treated *Enpp1*^*asj*^ (*triangles, n* = 8), and vehicle-treated *Enpp1*^*asj*^ mice (*circles*, *n* = 8). Dosing began on day 14. Treatment consisted of daily (qD) doses of 10 mg/kg (or 500 au/kg) of Enpp1-Fc formulated in phosphate-buffered saline (PBS) and weekly injections of GK1.5. Vehicle consisted of PBS supplemented with zinc and calcium. Deaths in the untreated *Enpp1*^*asj*^ cohort are denoted by red arrows. No deaths occurred in *Enpp1*^WT^ or the treated *Enpp1*^*asj*^ cohort. (*c*) Survival curves for Enpp1-Fc (*solid red line*) and vehicle-treated (*dashed black line*) *Enpp1*^*asj*^ mice; *p* = 0.003 (Mantle-Cox). (*d*) Plasma pyrophosphate (PP_i_) in *Enpp1*^WT^ and treated and untreated *Enpp1*^*asj*^ mice. The statistical significance between treated and untreated *Enpp1*^*asj*^ mice was *p* = 0.0015, Student’s two-tailed t test. Figure adapted from Reference 37.

Whatever pathological etiologies are driving the diverse phenotypes, the disruption of extracellular purinergic metabolism induced by ENPP1’s absence must account for the varied pathologic sequela. Supplying active ENPP1 to deficient patients using a protein replacement therapy strategy therefore constitutes a straightforward, tractable therapeutic approach, as demonstrated by the efficacy of an engineered biologic comprising the extracellular domain of ENPP1 fused to the Fc region of human immunoglobulin G1 (IgG1) (ENPP1-Fc) (Figure 8a) in the accepted murine model of GACI, *Enpp1^asj^* (Jackson Laboratory, stock number 012810). These mice were originally identified by the Jackson Laboratory in an ENU (N-ethyl-N-nitrosourea) screen and found to have an inactivating variant near the Enpp1 catalytic site at V246D, which misfolds the protein and reduces both protein expression and extracellular catalytic activity by ~75% (105). The mice developed a stiff posture due to calcifications of the joints and ligaments by approximately 2 months of age that is reminiscent of severely arthritic patients. However, unlike humans with biallelic inactivating ENPP1 variants, *Enpp1^asj^* mice do not spontaneously develop vascular calcifications unless placed on a diet high in phosphate and low in magnesium (acceleration diet) (50).

**Figure 9:**
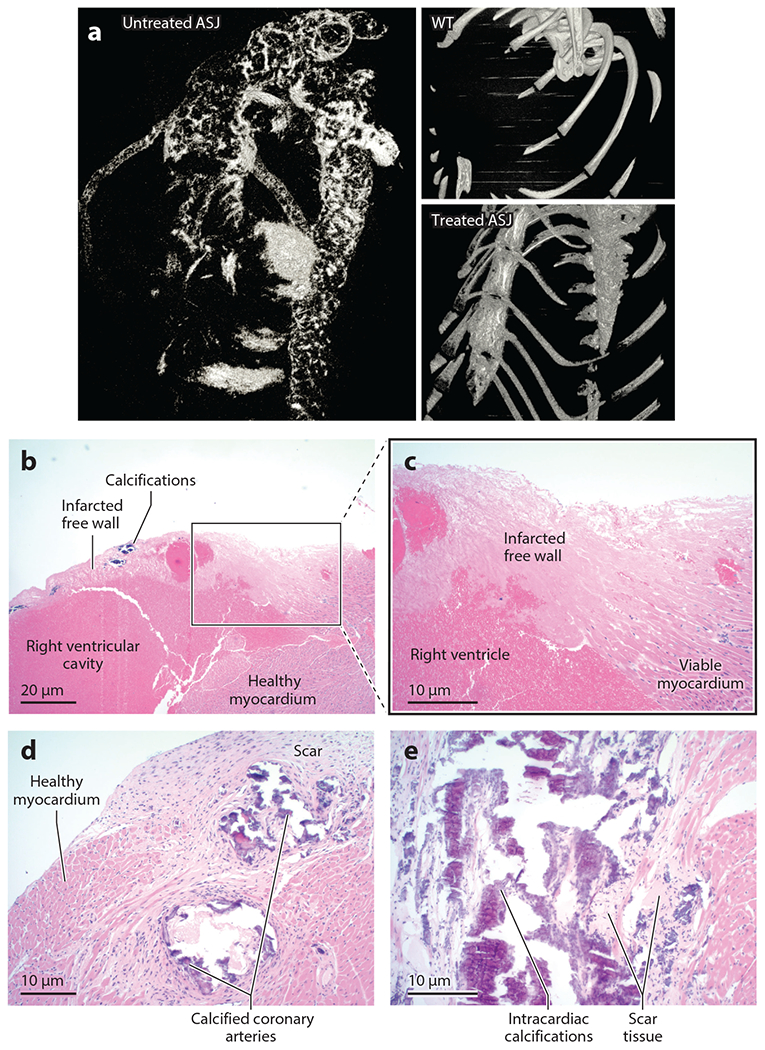
The efficacy of ectonucleotide pyrophosphatase/phosphodiesterase 1 (ENPP1) enzyme replacement in a murine model of generalized arterial calcification of infancy: histology. (*a*) Postmortem high-resolution microcomputed tomography scans revealed extensive calcifications in untreated *Enpp1*^*asj*^ mice in the hearts, coronary arteries, and ascending and descending aortas but absolutely no calcifications in these organs in the treated *Enpp1*^*asj*^ cohort or in *Enpp1*^WT^ mice. (*b*) Untreated *Enpp1*^*asj*^ mice, right ventricle [hematoxylin and eosin (H&E) stain]. Two untreated *Enpp1*^*asj*^ mice had large, confluent, myocardial infarctions in the free wall of the right ventricle. All treated *Enpp1*^*asj*^ mice displayed normal right ventricle myocardium (not shown). (*c*) Untreated *Enpp1*^*asj*^ mice, right ventricle (H&E), showing detail from the boxed area in panel *b*. (*d*) Untreated *Enpp1*^*asj*^ mice, coronary arteries (H&E). All untreated *Enpp1*^*asj*^ mice had coronary calcifications, with most displaying circumferential calcifications in coronary arteries surrounded by scar tissue. (*e*) Untreated *Enpp1*^*asj*^ mice, myocardial septum (H&E). Nearly all animals (77%) displayed intracardiac calcifications surrounded by scar tissue, as demonstrated in this animal in the myocardial septum. Figure adapted from Reference 37.

In proof-of-concept experiments testing the efficacy of ENPP1-Fc, heterozygous *Enpp1*^*asj*/+^ breeding pairs were placed on an acceleration diet. Weights of homozygous *Enpp1^asj^* progeny diverged from the WT siblings at day 26, when they exhibited a dramatic failure to thrive in which the animals lost weight, displayed progressive stiffness and reductions in physical activity, and soon expired with a median lifespan of 35 days (Figure 8b). In contrast, when daily subcutaneous doses of murine Enpp1-Fc at 10 mg/kg were given on postnatal day 14 to *Enpp1^asj^* mice on the acceleration diet, the mice gained weight comparable to that seen in the WT siblings, remained bright and active, and survived throughout a 55-day trial. The enzyme biologic was found to extend survival by eliminating cardiovascular calcifications and myocardial infarctions in the animals due to normalization of plasma PP_i_ in the dosed animals (Figures 8d and 9) (106).

A follow-on study performed in a second murine model of Enpp1 deficiency called *Enpp1^asj-2J^* mice confirmed and extended the above studies. *Enpp1^asj-2J^* mice have a large deletion in *Enpp1* (eliminating 93% of the coding region) and exhibit calcifications in the cardiovascular system (heart, aorta, carotid) on a regular chow diet, in contrast to *Enpp1^asj^* mice, which require the acceleration diet to induce cardiovascular calcifications. The use of *Enpp1^asj-2J^* mice therefore eliminated the potential confounding effects of the mineralization diet in the experimental design. *Enpp1^asj-2J^* mice placed on regular chow treated with weekly 10 mg/kg doses of human recombinant ENPP1-Fc showed >95% reductions in aorta calcification after 3 weeks of treatment. Moreover, terminal hemodynamics and echocardiography imaging at 6 weeks revealed normalization of the elevated arterial and left ventricular pressures, which translated into significant improvements in myocardial compliance, contractility, heart workload, and global cardiovascular efficiency. This study provided additional support that a protein replacement therapy in GACI would be effective at treating not just the vascular calcification but also the hypertension leading to cardiac failure in GACI patients (107).

A third preclinical study examined the effect of human ENPP1-Fc on arterial stenoses in *ttw* mice, as GACI induces not only severe calcification of the media of large and medium-sized arteries but also dramatic intimal proliferation leading to arterial stenoses within the first month of life (16, 108, 109). The intimal proliferation is thought to contribute to the hypertension, myocardial ischemia, and severe congestive heart failure in affected infants, placing them at significant risk of death within the first 6 months of life. This study demonstrated for the first time that enzyme replacement with human ENPP1-Fc can inhibit the accelerated vascular smooth muscle cell (VSMC) proliferation and arterial stenoses induced by ENPP1 deficiency. The inhibition of intimal VSMC proliferation resulted from the restoration of adenosine levels to normal values (via restoring cAMP as substrate for the adenosine producing enzyme CD73), demonstrating that in addition to PP_i_, other purinergic metabolites of ENPP1 (such as AMP and adenosine) are essential for maintaining physiologic vascular tone and, presumably, thrombosis (5).

**Figure 10:**
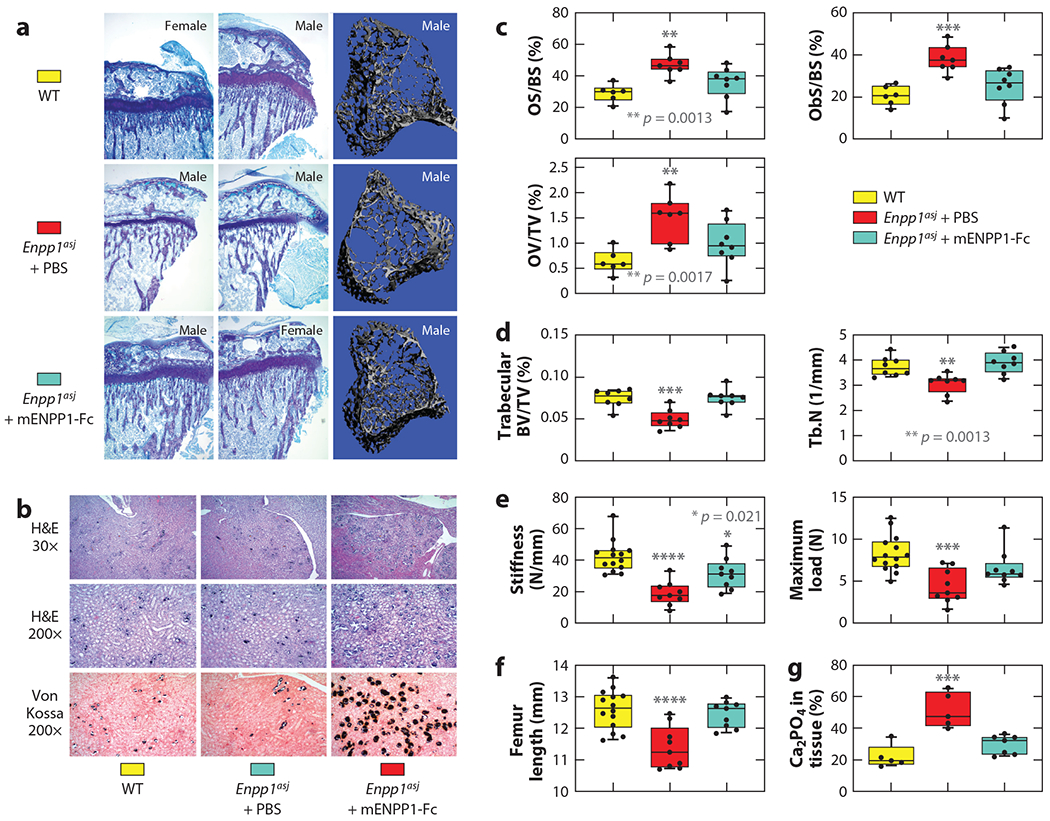
The effects of ectonucleotide pyrophosphatase/phosphodiesterase 1 (ENPP1) enzyme replacement on skeletal mineralization. (*a*) Proximal tibia histology of 5-week-old wild-type (WT) mice and mice on the acceleration diet treated between weeks 3 and 5 with vehicle [phosphate-buffered saline (PBS)] or murine Enpp1-Fc. Morphologically apparent reductions in trabecular bone and markedly thinner growth plates are noted in vehicle-treated *Enpp1*^*asj*^ mice, and Enpp1-Fc treatment markedly improved trabecular bone volume and growth plate thickness. (*b*) Vehicle-treated *Enpp1*^*asj*^ mice on the acceleration diet exhibited pronounced nephrocalcinosis throughout the renal parenchyma, including cortex and renal medulla, in contrast to Enpp1-Fc-treated *Enpp1*^*asj*^ mice. The calcification was most pronounced in renal tubules near the cortical-medullary junction. Top and middle: hematoxylin and eosin (H&E) stains; bottom: Von Kossa stains. (*c*) Histomorphometry of mice demonstrated reductions in osteomalacia in the murine Enpp1-Fc treated cohorts; osteoid surface area per bone surface area (OS/BS), osteoblast surface per bone surface (ObS/BS), and osteoid volume per total volume (OV/TV) (female mice shown). (*d*) Trabecular mineralization as measured by microcomputed tomography, (*e*) biomechanical properties, and (*f*) length of femurs demonstrates recovery of long bone mineralization in the Enpp1-Fc-treated cohort. Trabecular bone volume to total volume fraction (BV/TV), trabecular number (Tb.N), biomechanical stiffness (slope of the load versus displacement curve), and max load (also known as strength) are shown as tested in three-point bending until failure in male and female mice; 14 WT mice were treated with PBS(5F and 9M), 9 *Enpp1*^*asj*^ mice were treated with PBS (4F and 5M), and 9 *Enpp1*^*asj*^ mice were treated with mEnpp1-Fc (4F and 5 M). (*g*) Nephrocalcinosis, quantitated by a renal pathologist blinded to experimental groups, yielded no statistical difference in nephrocalcinosis between the WT and *Enpp1*^*asj*^ mice treated with mEnpp1-Fc, while vehicle-treated *Enpp1*^*asj*^ mice experienced approximately a twofold increase in nephrocalcinosis when compared with WT or mEnpp1-Fc-treated *Enpp1*^*asj*^ mice. Individual measurements are displayed as circles, with bar height representing the median and error bars denoting the interquartile range (25%–75%); *** indicates *p* < 0.001 and **** indicates *p* < 0.0001 (analysis of variance). Figure adapted from Reference 57.

Another preclinical study compared the skeletal phenotype in ARHR2 patients treated with conventional therapy to that of *Enpp1^asj^* mice on the acceleration diet treated with murine Enpp1-Fc. The study found that conventional treatment improved the rickets in ARHR2 patients but failed to correct the low BMD while also placing the patients at risk for medullary nephrocalcinosis (Figure 2c–j). Moreover, areal BMD by DXA (height-adjusted Z-scores at hip and total body less head sites) of an ARHR2 adolescent significantly decreased over a 2-year period while the patient was undergoing treatment with conventional therapy. In contrast, the lower trabecular and cortical bone mass as well as greater bone fragility were normalized and nephrocalcinosis was prevented in 5-week-old *Enpp1^asj^* mice with daily doses of 10 mg/kg recombinant murine Enpp1-Fc between weeks 2 and 5 (57) (Figure 10). These findings suggested that conventional ARHR2 therapy (calcitriol and phosphate) may not address the low bone mass present in ARHR2 patients while also placing individuals at risk for nephrocalcinosis. In contrast, ENPP1 enzyme replacement therapy corrected low bone mass and prevented renal calcifications in the murine model.

**Figure 11:**
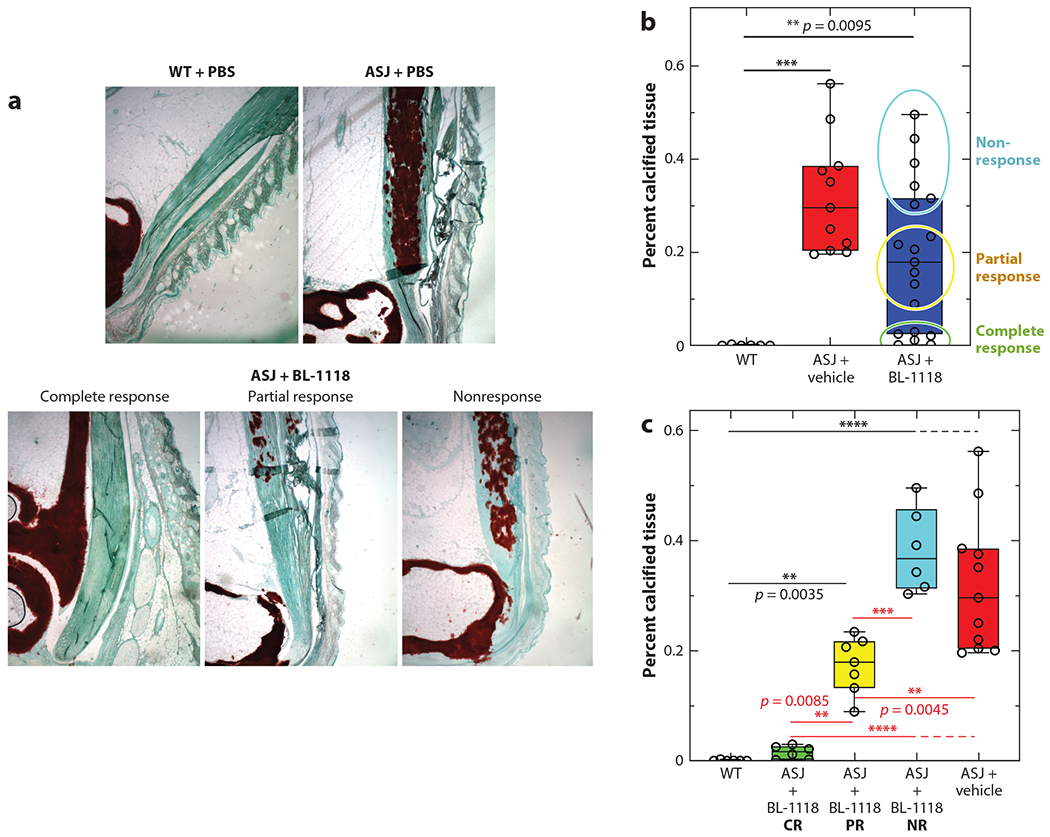
The efficacy of ectonucleotide pyrophosphatase/phosphodiesterase 1 (ENPP1) enzyme replacement in enthesopathy. (*a*) Representative histology of 23-week-old vehicle-treated wild-type (WT) and *Enpp1*^*asj*^ mice (*top*) compared with 23-week-old *Enpp1*^*asj*^ mice treated with weekly subcutaneous injections of 0.3 mg/kg BL-1118 chosen from complete responders, partial responders, and nonresponders (*bottom*). (*b*) Quantification of enthesis mineralization via Alizarin Red–stained sections in vehicle-treated WT and *Enpp1*^*asj*^ mice and BL-1118-treated *Enpp1*^*asj*^ mice. The data spread suggested grouping the response into complete responders, partial responders, and nonresponders (*circled data points*). (*c*) The same data as in panel *b* but grouped and analyzed into complete response (CR, 6 animals), partial response (PR, 7 animals), and nonresponse (NR, 6 animals). Black type and lines denote statistical significance between WT and treatment groups. Red type and lines denote statistical significance between treatment groups. Statistical significance is explicitly stated between 0.05 > *p* > 0.001; *** indicates *p* < 0.001 and **** indicates *p* < 0.0001 [analysis of variance (ANOVA) comparison of means]. Figure adapted from Reference 73.

A final preclinical study examined enthesis calcifications in *Enpp1^asj^* mice treated with a long-acting form of human ENPP1-Fc called BL-1118 optimized for potency and bioavailability using a combination of directed chemical evolution and glycopolishing techniques (110). *Enpp1^asj^* mice were fed a regular chow diet for 23 weeks and dosed between weeks 3 and 23 with vehicle or BL-1118 at 0.3 mg/kg. After 23 weeks, examination of the Achilles tendons revealed substantial calcifications in the tendons of the vehicle-treated mice and complete suppression of tendon calcification in one-third of the treated mice (6 of 19) as well as significant suppression in the tendon calcification in an additional one-third of the treated mice (7 of 19). PP_i_ levels increased in the treated *Enpp1^asj^* mice relative to their untreated siblings (1,358 ± 268 nM versus 51 ± 22 nM, respectively) but did not attain the levels observed in their WT siblings (2,235 ± 621 nM), suggesting that additional suppression of enthesis calcification might be possible at higher doses (73) (Figure 11 ).

## 7. BENCH TO BEDSIDE

There is a well-recognized and documented crisis in drug development, perhaps best described by Eroom’s law, which states that the number of new drugs approved per billion US dollars spent on R&D has halved roughly every 9 years since 1950, falling approximately 80-fold in inflation-adjusted terms between 1950 and 2010 (111). Approval rates for protein replacement therapies for monogenic disease are strikingly higher than that of biologics, small molecules, and grant-funded orphan drugs. The difference in success rates are stark—the likelihood of approval (LOA) from phase 1 through the US Food and Drug Administration basic license application approval for a protein replacement therapy reaching the clinic is 88%, and 91% if the therapy is first in class (112), compared with an LOA of 15% for all other molecular entities targeting all other disease entities (113). A shift in drug development toward biologics, and perhaps an increased capacity to translate human genetic data into disease pathogenesis, has likely been responsible for the improvement in the LOA of new drugs since 2010 (114). Importantly, because rare diseases unveil biological pathways frequently disrupted in more common disorders affecting the general medical population, expansion of rare disease therapeutics is the rule rather than the exception. These facts have steered the funding landscape for rare disease toward venture capital entities seeking novel therapeutics with a high likelihood of approval in severe diseases without existing, effective treatment.

Inozyme Pharma was founded in 2017 to translate ENPP1 enzyme replacement into patients with GACI and other mineralization disorders. The half-life of the initial murine Enpp1-Fc biologic in mice was 6 h (106), and the half-life of the human GMP biologic manufactured by Inozyme (INZ-701) was 35 h in mice (115). Preclinical studies of INZ-701 in *Enpp1^asj^* mice dosed between 0.2 and 5 mg/kg every other day restored circulating levels of PP_i_ and prevented pathological calcification in all the tested organs, restored growth parameters, corrected bone defects, improved clinical signs, and decreased mortality, demonstrating the potential of INZ-701 to treat ENPP1 deficiency in humans (115). Clinical trials of INZ-701 began in GACI and PXE patients in January 2021 (ClinicalTrials.gov NCT04686175 and NCT05030831), the results of which found the drug to be well tolerated and with a favorable safety profile. The half-life of INZ-701 in humans is 125 h, and twice weekly doses of INZ-701 at 0.2, 0.6, and 1.8 mg/kg resulted in rapid, significant, and sustained increase plasma PP_i_ in GACI at all doses and in PXE patients at 1.8 mg/kg (116). The favorable phase 1/2 results support extension into infants with GACI (ClinicalTrials.gov NCT05734196) with the hope of advancement to phase 3 pivotal trials in 2023.

## 8. CONCLUDING REMARKS

As with other rare bone diseases (117), lessons learned in patients with ENPP1 deficiency are likely to inform the treatment of patients in the general medical population. Progressive mineralization of vascular and soft tissues accompanied by increasingly reduced skeletal mineralization is referred to as a paradoxical mineralization disorder to emphasize the opposing tissue-specific processes and confusing pathogenesis. Paradoxical mineralization also occurs in the general medical population in aging adults (118, 119) and in patients with a syndrome known as chronic kidney disease and bone mineralization disorder (CKD-MBD), who exhibit bone that inversely correlates with vascular calcification (120, 121) as well as markedly reduced plasma PP_i_ (122, 123). Moreover, the high fracture risk and associated mortality in CKD-MBD patients has not substantially improved over the last 20 years despite significant progress in other forms of osteoporosis (121). It remains to be seen whether an understanding of the opposing roles of ENPP1 in the suppression of ectopic mineralization and maintenance of bone mass will unlock the treatment of CKD-MBD patients, but efforts to this end are currently being explored, including a natural history study in calciphylaxis patients demonstrating that their plasma PP_i_ levels are comparable to levels observed in GACI patients and correlate with mortality. Moreover, the recent understanding of catalysis-independent signaling effects of ENPP1 will motivate the exploration of bone targeting therapeutics on skeletal phenotypes induced by ENPP1 deficiency. It is hoped that the result of these efforts will be the development of effective therapeutics not only for patients suffering from increased ectopic calcification in the vascular and soft tissue but also for those with concurrently reduced skeletal mineralization as seen in the paradoxical mineralization disorders of CKD-MBD and aging.

## Figures and Tables

**Table 1:** Genetic diseases due to disruptions in the extracellular purine metabolic pathway

Disease	Variant protein(s)	Relevant metabolite(s)	Phenotype(s)	Reference(s)
Pseudoxanthoma elasticum (PXE)	ABCC6	ATP	Skin and retinal calcifications; retinal detachment; blindness; slowly progressing vascular calcifications	9
Generalized calcification of infancy (GACI)	ENPP1 and ABCC6	PP_i_	Intrauterine and infantile calcifications of the large arteries; periarticular calcifications; infantile stroke, severe hypertension, and cardiac failure	10
Autosomal recessive hypophosphatemic rickets type 2 (ARHR2)	ENPP1	PP_i_ and P_i_	Phosphate wasting rickets; progressive enthesopathies; hearing loss	11, 12
Hypophosphatasia (HPP)	TNAP	P_i_	Poor skeletal mineralization resulting in hypoxia in infants; poor dentition	13
Arterial calcifications due to CD73 deficiency (AC/DC)	CD73	AMP/adenosine	Calcifications of arteries in the lower extremities; periarticular calcifications of joints	14

Abbreviations: ABCC6, ATP-binding cassette subfamily C member 6; AMP, adenosine monophosphate; ATP, adenosine 5′-triphosphate; CD73, 5′-nucleotidase; ENPP1, ectonucleotide pyrophosphatase/phosphodiesterase 1; P_i_, phosphate; PP_i_, pyrophosphate; TNAP, tissue-nonspecific alkaline phosphatase.

**Table 2:** Transcriptome changes induced by ENPP1 deficiency

Gene family	Gene	Regulation and method
**Bone**		
Wnt activity	*Lrp1* *	↓ RNAseq
	*Wnt10b* **	↓ RNAseq
	*Wnt16* ****	↓ RNAseq
Collagens	Col3a1*, Col6a2**, Col6a3**, Col8a2****, Col12a1****, Col16a1**	↓ RNAseq
Bone formation	*Bglap* (OCN)**	−2.6** qPCR
	*Ibsp* (BSP)*	−2.1* qPCR
**Liver via qPCR**		
Wnt activity	*Sfrp1* *	+2.1*
	*Fzd8* *	−3.4**
**Kidney via qPCR**		
Wnt activity	*Wif1* *	+11.7*

RNAseq and qPCR of RNA extracted from whole bone, liver, and kidney of male *Enpp1*^*asj/asj*^ mice and WT siblings (*n* = 4, each cohort). Affected members are included on the basis of significant differential gene expression in 10- or 23-week-old animals. RNAseq adjusted p-values:

* p_adj_ < 0.05,

** p_adj_ < 0.01,

*** p_adj_ < 0.005,

**** p_adj_ < 0.001. qPCR Student’s t test:

* p < 0.05,

** p < 0.01.

Abbreviations: qPCR, quantitative polymerase chain reaction; RNAseq, RNA sequencing; WT, wild type.

## SUMMARY POINTS

1. Ectonucleotide pyrophosphatase/phosphodiesterase 1 (ENPP1) deficiency may induce life-threatening vascular calcifications and arterial stenosis in infants with generalized arterial calcification of infancy (GACI).
2. GACI manifests as early as the second trimester, and infants frequently present as in crisis within the first few weeks of life.
3. By 6 months of age, approximately 50% of GACI infants succumb to disease, and those who survive are stabilized but invariably have elevated fibroblast growth factor 23 (FGF23) and develop a severe phosphate (P_i_) wasting rickets called autosomal recessive hypophosphatemic rickets type 2 (ARHR2).
4. The ARHR2 phenotype may be an adaptive response to low plasma pyrophosphate (PP_i_) levels, in which the organism attempts to rebalance the plasma P_i_/PP_i_ ratio at the expense of the skeleton.
5. Individuals with heterozygous pathogenic ENPP1 variants may exhibit low bone mass manifesting as early-onset osteoporosis and painful calcifications of tendons.
6. Heterozygous pathogenic variants of ENPP1 have been observed in patients with spinal enthesopathies in diseases called ossification of the posterior longitudinal ligament (OPLL) and dystrophic idiopathic spinal hyperostosis (DISH).
7. The vascular calcifications in GACI are frequently treated with bisphosphonates, which have recently been found to be without significant clinical benefit if administered later than the first week of life.
8. Rickets present in ARHR2 can be effectively treated with supplemental phosphate and calcitriol, but limitations of this therapy include an increased risk for nephrocalcinosis, as well as an unclear benefit in terms of addressing the low bone mass.

## FUTURE ISSUES

1. Enzyme replacement therapy (ERT) using a soluble ectonucleotide pyrophosphatase/phosphodiesterase 1 (ENPP1) enzyme fused to an immunoglobulin G1 (IgG1) Fc domain effectively suppressed vascular calcifications, disease sequela, and death in a murine model of generalized arterial calcification of infancy (GACI).
2. ENPP1 ERT also effectively suppressed intimal hyperplasia, improved cardiovascular function, and partially prevented enthesis calcification in ENPP1-deficient mice.
3. Phase 1/2 data in humans with ENPP1 and ABCC6 deficiency found the therapy to be well tolerated with a favorable safety profile. Twice weekly doses of the biologic at 1.8 mg/kg normalized plasma pyrophosphate levels in both autosomal recessive hypophosphatemic rickets type 2 and pseudoxanthoma elasticum patients.
4. Clinical trials of ENPP1 ERT in infants with GACI are currently underway.
5. ENPP1 therapy may be helpful in patients with mineralization disorders associated with spinal hyperostosis, such as ossification of the posterior longitudinal ligament and chronic kidney disease and bone mineralization disorder.

## References

[1] Zalatan JG, Fenn TD, Brunger AT, Herschlag D. 2006. Structural and functional comparisons of nucleotide pyrophosphatase/phosphodiesterase and alkaline phosphatase: implications for mechanism and evolution. Biochemistry 45(32):9788–803 16893180 10.1021/bi060847t

[2] Li L, Yin Q, Kuss P, Maliga Z, Millan JL, Wu H, Mitchison TJ. 2014. Hydrolysis of 2′3′-cGAMP by ENPP1 and design of nonhydrolyzable analogs. Nat. Chem. Biol 10(12):1043–48 25344812 10.1038/nchembio.1661 PMC4232468

[3] Fleisch H, Bisaz S. 1962. Mechanism of calcification: inhibitory role of pyrophosphate. Nature 195:911 10.1038/195911a0 13893487

[4] Meyer JL. 1984. Can biological calcification occur in the presence of pyrophosphate? Arch. Biochem. Biophys 231(1):1–8 6326671 10.1016/0003-9861(84)90356-4

[5] Nitschke Y, Yan Y, Buers I, Kintziger K, Askew K, Rutsch F. 2018. ENPP1-Fc prevents neointima formation in generalized arterial calcification of infancy through the generation of AMP. Exp. Mol. Med 50(10):139 30369595 10.1038/s12276-018-0163-5 PMC6204430

[6] Kato K, Nishimasu H, Oikawa D, Hirano S, Hirano H, 2018. Structural insights into cGAMP degradation by ecto-nucleotide pyrophosphatase phosphodiesterase 1. Nat. Commun 9(1):4424 30356045 10.1038/s41467-018-06922-7 PMC6200793

[7] Carozza JA, Cordova AF, Brown JA, AlSaif Y, Bohnert V, 2022. ENPP1’s regulation of extracellular cGAMP is a ubiquitous mechanism of attenuating STING signaling. PNAS 119(21):e2119189119 35588451 10.1073/pnas.2119189119 PMC9173814

[8] Jackson EK, Dubey RK. 2001. Role of the extracellular cAMP-adenosine pathway in renal physiology. Am. J. Physiol. Renal Physiol 281(4):F597–612 11553506 10.1152/ajprenal.2001.281.4.F597

[9] Bergen AA, Plomp AS, Schuurman EJ, Terry S, Breuning M, 2000. Mutations in *ABCC6* cause pseudoxanthoma elasticum. Nature Genet. 25(2):228–31 10835643 10.1038/76109

[10] Rutsch F, Vaingankar S, Johnson K, Goldfine I, Maddux B, 2001. PC-1 nucleoside triphosphate pyrophosphohydrolase deficiency in idiopathic infantile arterial calcification. Am. J. Pathol 158(2):543–54 11159191 10.1016/S0002-9440(10)63996-X PMC1850320

[11] Levy-Litan V, Hershkovitz E, Avizov L, Leventhal N, Bercovich D, 2010. Autosomal-recessive hypophosphatemic rickets is associated with an inactivation mutation in the *ENPP1* gene. Am. J. Hum. Genet 86(2):273–78 20137772 10.1016/j.ajhg.2010.01.010 PMC2820183

[12] Lorenz-Depiereux B, Schnabel D, Tiosano D, Hausler G, Strom TM. 2010. Loss-of-function *ENPP1* mutations cause both generalized arterial calcification of infancy and autosomal-recessive hypophosphatemic rickets. Am. J. Hum. Genet 86(2):267–72 20137773 10.1016/j.ajhg.2010.01.006 PMC2820166

[13] Rathbun JC. 1948. Hypophosphatasia; a new developmental anomaly. Am. J. Dis. Child 75(6):822–31 10.1001/archpedi.1948.02030020840003 18110134

[14] St Hilaire C, Ziegler SG, Markello TC, Brusco A, Groden C, 2011. *NT5E* mutations and arterial calcifications. New Engl. J. Med 364(5):432–42 21288095 10.1056/NEJMoa0912923 PMC3049958

[15] Durante G. 1899. Athérome Congénital de l’aorte et de l’artère Pulmonaire. Bull. Soc. Anat 74:97–101

[16] Bryant J, White W. 1901. A case of calcification of the arteries and obliterative endarteritis, associated with hydronephrosis, in a child aged six months. Guy’s Hospital Rep. 55:17–28

[17] Stuart G, Wren C, Bain H. 1990. Idiopathic infantile arterial calcification in two siblings: failure of treatment with diphosphonate. Br. Heart J 64(2):156–59 2118367 10.1136/hrt.64.2.156 PMC1024357

[18] Fleisch H, Maerki J, Russell RG. 1966. Effect of pyrophosphate on dissolution of hydroxyapatite and its possible importance in calcium homeostasis. Proc. Soc. Exp. Biol. Med 122(2):317–20 4292457 10.3181/00379727-122-31123

[19] Fleisch H, Russell RG, Straumann F. 1966. Effect of pyrophosphate on hydroxyapatite and its implications in calcium homeostasis. Nature 212(5065):901–3 4306793 10.1038/212901a0

[20] Meradji M, de Villeneuve VH, Huber J, de Bruijn WC, Pearse RG. 1978. Idiopathic infantile arterial calcification in siblings: radiologic diagnosis and successful treatment. J. Pediatr 92(3):401–5 416189 10.1016/s0022-3476(78)80427-2

[21] Stuart AG. 1993. Idiopathic arterial calcification of infancy and pyrophosphate deficiency. J. Pediatr 123(1):170–71 8391568 10.1016/s0022-3476(05)81575-6

[22] Sholler GF, Yu JS, Bale PM, Hawker RE, Celermajer JM, Kozlowski K. 1984. Generalized arterial calcification of infancy: three case reports, including spontaneous regression with long-term survival. J. Pediatr 105(2):257–60 6747757 10.1016/s0022-3476(84)80123-7

[23] Ciana G, Trappan A, Bembi B, Benettoni A, Maso G, 2006. Generalized arterial calcification of infancy: two siblings with prolonged survival. Eur.J. Pediatr 165(4):258–63 16315058 10.1007/s00431-005-0035-6

[24] Marrott PK, Newcombe KD, Becroft DM, Friedlander DH. 1984. Idiopathic infantile arterial calcification with survival to adult life. Pediatr. Cardiol 5(2):119–22 6473121 10.1007/BF02424963

[25] Thiaville A, Smets A, Clercx A, Perlmutter N. 1994. Idiopathic infantile arterial calcification: a surviving patient with renal artery stenosis. Pediatr. Radiol 24(7):506–8 7885787 10.1007/BF02015014

[26] Nitschke Y, Baujat G, Botschen U, Wittkampf T, du Moulin M, 2012. Generalized arterial calcification of infancy and pseudoxanthoma elasticum can be caused by mutations in either *ENPP1* or *ABCC6*. Am. J. Hum. Genet 90(1):25–39 22209248 10.1016/j.ajhg.2011.11.020 PMC3257960

[27] Ferreira CR, Hackbarth ME, Ziegler SG, Pan KS, Roberts MS, 2020. Prospective phenotyping of long-term survivors of generalized arterial calcification of infancy (GACI). Genet. Med 23(2):396–407 33005041 10.1038/s41436-020-00983-0 PMC7867608

[28] Ferreira CR, Kintzinger K, Hackbarth ME, Botschen U, Nitschke Y, 2021. Ectopic calcification and hypophosphatemic rickets: natural history of ENPP1 and ABCC6 deficiencies. J. Bone Miner. Res 36(11):2193–202 34355424 10.1002/jbmr.4418 PMC8595532

[29] Johnson K, Moffa A, Chen Y, Pritzker K, Goding J, Terkeltaub R. 1999. Matrix vesicle plasma cell membrane glycoprotein-1 regulates mineralization by murine osteoblastic MC3T3 cells. J. Bone Miner. Res 14(6):883–92 10352096 10.1359/jbmr.1999.14.6.883

[30] Hosoda Y, Yoshimura Y, Higaki S. 1981. A new breed of mouse showing multiple osteochondral lesions—twy mouse. Ryumachi 21(Suppl.):157–64 7344126

[31] Sakamoto M, Hosoda Y, Kojimahara K, Yamazaki T, Yoshimura Y. 1994. Arthritis and ankylosis in twy mice with hereditary multiple osteochondral lesions: with special reference to calcium deposition. Pathol. Int 44(6):420–27 8055108 10.1111/j.1440-1827.1994.tb01705.x

[32] Okawa A, Nakamura I, Goto S, Moriya H, Nakamura Y, Ikegawa S. 1998. Mutation in *Npps* in a mouse model of ossification of the posterior longitudinal ligament of the spine. Nat. Genet 19(3):271–73 9662402 10.1038/956

[33] Kobayashi Y, Goto S, Tanno T, Yamazaki M, Moriya H. 1998. Regional variations in the progression of bone loss in two different mouse osteopenia models. Calcif. Tissue Int 62(5):426–36 9541520 10.1007/s002239900455

[34] Okawa A, Goto S, Moriya H. 1999. Calcitonin simultaneously regulates both periosteal hyperostosis and trabecular osteopenia in the spinal hyperostotic mouse (twy/twy) in vivo. Calcif. Tissue Int 64(3):239–47 10024383 10.1007/s002239900610

[35] Jansen S, Perrakis A, Ulens C, Winkler C, Andries M, 2012. Structure of NPP1, an ectonucleotide pyrophosphatase/phosphodiesterase involved in tissue calcification. Structure 20(11):1948–59 23041369 10.1016/j.str.2012.09.001

[36] Hausmann J, Kamtekar S, Christodoulou E, Day JE, Wu T, 2011. Structural basis of substrate discrimination and integrin binding by autotaxin. Nat. Struct. Mol. Biol 18(2):198–204 21240271 10.1038/nsmb.1980 PMC3064516

[37] Albright RA, Ornstein DL, Cao W, Chang WC, Robert D, 2014. Molecular basis of purinergic signal metabolism by ectonucleotide pyrophosphatase/phosphodiesterases 4 and 1 and implications in stroke. J. Biol. Chem 289(6):3294–306 24338010 10.1074/jbc.M113.505867 PMC3916532

[38] Gorelik A, Randriamihaja A, Illes K, Nagar B. 2017. A key tyrosine substitution restricts nucleotide hydrolysis by the ectoenzyme NPP5. FEBS J. 284(21):3718–26 28898552 10.1111/febs.14266

[39] Morita J, Kano K, Kato K, Takita H, Sakagami H, 2016. Structure and biological function of ENPP6, a choline-specific glycerophosphodiester-phosphodiesterase. Sci. Rep 6:20995 26888014 10.1038/srep20995 PMC4757880

[40] Gorelik A, Liu F, Illes K, Nagar B. 2017. Crystal structure of the human alkaline sphingomyelinase provides insights into substrate recognition. J. Biol. Chem 292(17):7087–94 28292932 10.1074/jbc.M116.769273 PMC5409475

[41] O’Neill WC, Lomashvili KA, Malluche HH, Faugere MC, Riser BL. 2011. Treatment with pyrophosphate inhibits uremic vascular calcification. Kidney Int. 79(5):512–17 21124302 10.1038/ki.2010.461 PMC3183997

[42] Francis MD. 1969. The inhibition of calcium hydroxypatite crystal growth by polyphosphonates and polyphosphates. Calcif. Tissue Res 3(2):151–62 5769901 10.1007/BF02058658

[43] Francis MD, Russell RG, Fleisch H. 1969. Diphosphonates inhibit formation of calcium phosphate crystals in vitro and pathological calcification in vivo. Science 165(3899):1264–66 4308521 10.1126/science.165.3899.1264

[44] Fleisch HA, Russell RG, Bisaz S, Muhlbauer RC, Williams DA. 1970. The inhibitory effect of phosphonates on the formation of calcium phosphate crystals in vitro and on aortic and kidney calcification in vivo. Eur. J. Clin. Investig 1(1):12–18 4319371 10.1111/j.1365-2362.1970.tb00591.x

[45] Hansen NM, Felix R, Bisaz S, Fleisch H. 1976. Aggregation of hydroxyapatite crystals. Biochim. Biophys. Acta Gen. Subj 451(2):549–59 10.1016/0304-4165(76)90150-1 826271

[46] Rutsch F, Boyer P, Nitschke Y, Ruf N, Lorenz-Depierieux B, 2008. Hypophosphatemia, hyperphosphaturia, and bisphosphonate treatment are associated with survival beyond infancy in generalized arterial calcification of infancy. Circ. Cardiovasc. Genet 1(2):133–40 20016754 10.1161/CIRCGENETICS.108.797704 PMC2794045

[47] Villa-Bellosta R, Sorribas V. 2013. Prevention of vascular calcification by polyphosphates and nucleotides—role of ATP. Circ. J 77(8):2145–51 23595088 10.1253/circj.cj-13-0016

[48] Hollwey A, Forster C, Mushtaq T. 2019. Use of disodium etidronate and sodium thiosulfate in a premature neonate with generalised arterial calcification of infancy. Arch. Dis. Child 104:e2 (Abstr.). 10.1136/archdischild-2019-nppc.41

[49] Omarjee L, Nitschke Y, Verschuere S, Bourrat E, Vignon MD, 2020. Severe early-onset manifestations of pseudoxanthoma elasticum resulting from the cumulative effects of several deleterious mutations in *ENPP1, ABCC6* and *HBB*: transient improvement in ectopic calcification with sodium thiosulfate. Br. J. Dermatol 183(2):367–72 31646622 10.1111/bjd.18632

[50] Kingman J, Uitto J, Li Q. 2017. Elevated dietary magnesium during pregnancy and postnatal life prevents ectopic mineralization in *Enpp1^asj^* mice, a model for generalized arterial calcification of infancy. Oncotarget 8(24):38152–60 28402956 10.18632/oncotarget.16687 PMC5503522

[51] Luo H, Li Q, Cao Y, Uitto J. 2020. Therapeutics development for pseudoxanthoma elasticum and related ectopic mineralization disorders: update 2020. J. Clin. Med 10(1):114 33396306 10.3390/jcm10010114 PMC7795895

[52] Dursun F, Atasoy Ozturk T, Guven S, Kirmizibekmez H, Seymen Karabulut G, 2019. Magnesium and anti-phosphate treatment with bisphosphonates for generalised arterial calcification of infancy: a case report. J. Clin. Res. Pediatr. Endocrinol 11(3):311–18 30525344 10.4274/jcrpe.galenos.2018.2018.0204 PMC6745454

[53] Otero JE, Gottesman GS, McAlister WH, Mumm S, Madson KL, 2013. Severe skeletal toxicity from protracted etidronate therapy for generalized arterial calcification of infancy. J. Bone Miner. Res 28(2):419–30 22972716 10.1002/jbmr.1752

[54] Theng EH, Brewer CC, Oheim R, Zalewski CK, King KA, 2022. Characterization of hearing-impairment in generalized arterial calcification of infancy (GACI). Orphanet J. Rare Dis 17(1):273 35854274 10.1186/s13023-022-02410-w PMC9295326

[55] Maulding ND, Kavanagh D, Zimmerman K, Coppola G, Carpenter TO, 2020. Genetic pathways disrupted by ENPP1 deficiency provide insight into mechanisms of osteoporosis, osteomalacia, and paradoxical mineralization. Bone 142:115656 32980560 10.1016/j.bone.2020.115656 PMC7744330

[56] Ferreira CR, Ziegler SG, Gupta A, Groden C, Hsu KS, Gahl WA. 2016. Treatment of hypophosphatemic rickets in generalized arterial calcification of infancy (GACI) without worsening of vascular calcification. Am. J. Med. Genet. A 170(5):1308–11 10.1002/ajmg.a.37574 PMC4833596 26857895

[57] Ferreira CR, Kavanagh D, Oheim R, Zimmerman K, Sturznickel J, 2021. Response of the ENPP1-deficient skeletal phenotype to oral phosphate supplementation and/or enzyme replacement therapy: comparative studies in humans and mice. J. Bone Miner. Res 36(5):942–55 33465815 10.1002/jbmr.4254 PMC8739051

[58] Erben RG. 2018. Physiological actions of fibroblast growth factor-23. Front. Endocrinol 9:267 10.3389/fendo.2018.00267 PMC5985418 29892265

[59] Stern R, Levi DS, Gales B, Rutsch F, Salusky IB. 2021. Correspondence on “Prospective phenotyping of long-term survivors of generalized arterial calcification of infancy (GACI)” by Ferreira et al Genet. Med 23(10):2006–7 34127825 10.1038/s41436-021-01228-4

[60] Lomashvili KA, Garg P, Narisawa S, Millan JL, O’Neill WC. 2008. Upregulation of alkaline phosphatase and pyrophosphate hydrolysis: potential mechanism for uremic vascular calcification. Kidney Int. 73(9):1024–30 18288101 10.1038/ki.2008.26 PMC3010853

[61] Sheen CR, Kuss P, Narisawa S, Yadav MC, Nigro J, 2015. Pathophysiological role of vascular smooth muscle alkaline phosphatase in medial artery calcification. J. Bone Miner. Res 30(5):824–36 25428889 10.1002/jbmr.2420 PMC4406354

[62] Murshed M, Harmey D, Millan JL, McKee MD, Karsenty G. 2005. Unique coexpression in osteoblasts of broadly expressed genes accounts for the spatial restriction of ECM mineralization to bone. Genes Dev. 19(9):1093–104 15833911 10.1101/gad.1276205 PMC1091743

[63] Shaw HM, Benjamin M. 2007. Structure-function relationships of entheses in relation to mechanical load and exercise. Scand. J. Med. Sci. Sports 17(4):303–15 17490450 10.1111/j.1600-0838.2007.00689.x

[64] Benjamin M, Evans EJ. 1990.Fibrocartilage. J. Anat 171:1–15 2081696 PMC1257123

[65] Benjamin M, Ralphs JR. 1998. Fibrocartilage in tendons and ligaments—an adaptation to compressive load. J. Anat 193(Part 4):481–94 10029181 10.1046/j.1469-7580.1998.19340481.x PMC1467873

[66] Benjamin M, Rufai A, Ralphs JR. 2000. The mechanism of formation of bony spurs (enthesophytes) in the Achilles tendon. Arthritis Rheum. 43(3):576–83 10728751 10.1002/1529-0131(200003)43:3<576::AID-ANR14>3.0.CO;2-A

[67] Hardy DC, Murphy WA, Siegel BA, Reid IR, Whyte MP 1989. X-linked hypophosphatemia in adults: prevalence of skeletal radiographic and scintigraphic features. Radiology 171(2):403–14 2539609 10.1148/radiology.171.2.2539609

[68] Reid IR, Hardy DC, Murphy WA, Teitelbaum SL, Bergfeld MA, Whyte MP. 1989. X-linked hypophosphatemia: a clinical, biochemical, and histopathologic assessment of morbidity in adults. Medicine 68(6):336–52 2811660

[69] Chalmers J. 1993. Enthesopathy as the presenting feature of X-linked hypophosphatemia. A case report. Acta Orthop. Scand 64(2):221–23 8498190 10.3109/17453679308994575

[70] Liang G, Katz LD, Insogna KL, Carpenter TO, Macica CM. 2009. Survey of the enthesopathy of X-linked hypophosphatemia and its characterization in Hyp mice. Calcif. Tissue Int 85(3):235–46 19609735 10.1007/s00223-009-9270-6 PMC2988401

[71] Ramonda R, Sfriso P, Podswiadek M, Oliviero F, Valvason C, Punzi L. 2005. The enthesopathy of vitamin D-resistant osteomalacia in adults. Reumatismo 57(1):52–56 15776147 10.4081/reumatismo.2005.52

[72] Karaplis AC, Bai X, Falet JP, Macica CM. 2012. Mineralizing enthesopathy is a common feature of renal phosphate-wasting disorders attributed to FGF23 and is exacerbated by standard therapy in Hyp mice. Endocrinology 153(12):5906–17 23038738 10.1210/en.2012-1551 PMC3512070

[73] Ferreira CR, Ansh AJ, Nester C, O’Brien C, Stabach PR, 2022. Musculoskeletal comorbidities and quality of life in ENPP1-deficient adults and the response of enthesopathy to enzyme replacement therapy in murine models. J. Bone Miner. Res 37(3):494–504 34882836 10.1002/jbmr.4487 PMC9667476

[74] Masel JP, Cartwright DW, Latham SC. 1981. Hypophosphataemic vitamin D-resistant rickets—a cause of spinal stenosis in adults. Australas. Radiol 25(3):264–71 6284105 10.1111/j.1440-1673.1981.tb02259.x

[75] van der Kraan PM, van den Berg WB. 2007. Osteophytes: relevance and biology. Osteoarthritis Cartilage 15(3):237–44 17204437 10.1016/j.joca.2006.11.006

[76] Rogers J, Shepstone L, Dieppe P 2004. Is osteoarthritis a systemic disorder of bone? Arthritis Rheum 50(2):452–57 14872487 10.1002/art.20136

[77] Gafni R, Spector E, Hartley I, Redd B, Mitnik G, Collins M. 2020. Enthesophytes are a common feature of FGF23-mediated hypophosphatemia due to tumor-induced osteomalacia. J. Endocr. Soc 4(Suppl. 1):A487 (Abstr.). 10.1210/jendso/bvaa046.960

[78] Kawaguchi Y, Nakano M, Yasuda T, Seki S, Suzuki K, 2017. Serum biomarkers in patients with ossification of the posterior longitudinal ligament (OPLL): inflammation in OPLL. PLOS ONE 12(5):e0174881 28467440 10.1371/journal.pone.0174881 PMC5414934

[79] Kawaguchi Y, Kitajima I, Nakano M, Yasuda T, Seki S, 2019. Increase of the serum FGF-23 in ossification of the posterior longitudinal ligament. Global Spine J. 9(5):492–98 31431871 10.1177/2192568218801015 PMC6686384

[80] Mader R, Verlaan JJ, Buskila D. 2013. Diffuse idiopathic skeletal hyperostosis: clinical features and pathogenic mechanisms. Nat. Rev. Rheumatol 9(12):741–50 24189840 10.1038/nrrheum.2013.165

[81] Nishimura S, Nagoshi N, Iwanami A, Takeuchi A, Hirai T, 2018. Prevalence and distribution of diffuse idiopathic skeletal hyperostosis on whole-spine computed tomography in patients with cervical ossification of the posterior longitudinal ligament: a multicenter study. Clin. Spine Surg 31(9):E460–65 30113323 10.1097/BSD.0000000000000701

[82] Murakami Y, Mashima N, Morino T, Fukuda T, Iwase M, 2019. Association between vertebral fracture and diffuse idiopathic skeletal hyperostosis. Spine 44(18):E1068–74 31479433 10.1097/BRS.0000000000003151

[83] Park S, Lee DH, Ahn J, Cho JH, Lee SK, 2020. How does ossification of posterior longitudinal ligament progress in conservatively managed patients? Spine 45(4):234–43 31513119 10.1097/BRS.0000000000003240

[84] Koshizuka Y, Kawaguchi H, Ogata N, Ikeda T, Mabuchi A, 2002. Nucleotide pyrophosphatase gene polymorphism associated with ossification of the posterior longitudinal ligament of the spine. J. Bone Miner Res 17(1):138–44 11771660 10.1359/jbmr.2002.17.1.138

[85] Nakajima M, Takahashi A, Tsuji T, Karasugi T, Baba H, 2014. A genome-wide association study identifies susceptibility loci for ossification of the posterior longitudinal ligament of the spine. Nature Genet. 46(9):1012–16 25064007 10.1038/ng.3045

[86] Karasugi T, Nakajima M, Ikari K, Genet. Study Group Investig. Comm. Ossification Spinal Ligaments, Tsuji T, 2013. A genome-wide sib-pair linkage analysis of ossification of the posterior longitudinal ligament of the spine. J. Bone Miner. Metabol 31(2):136–43 10.1007/s00774-012-0404-y 23138351

[87] Mader R, Pappone N, Baraliakos X, Eshed I, Sarzi-Puttini P, 2021. Diffuse idiopathic skeletal hyperostosis (DISH) and a possible inflammatory component. Curr. Rheumatol. Rep 23(1):6 33496875 10.1007/s11926-020-00972-x

[88] Chen J, Song D, Wang X, Shen X, Li Y, Yuan W 2011. Is ossification of posterior longitudinal ligament an enthesopathy? Int. Orthop 35(10):1511–16 21104248 10.1007/s00264-010-1163-9 PMC3174289

[89] Kato H, Ansh AJ, Lester ER, Kinoshita Y, Hidaka N, 2022. Identification of ENPP1 haploinsufficiency in patients with diffuse idiopathic skeletal hyperostosis and early-onset osteoporosis. J. Bone Miner. Res 37(6):1125–35 35340077 10.1002/jbmr.4550 PMC9177665

[90] Oheim R, Zimmerman K, Maulding ND, Sturznickel J, von Kroge S, 2020. Human heterozygous ENPP1 deficiency is associated with early onset osteoporosis, a phenotype recapitulated in a mouse model of Enpp1 deficiency. J. Bone Miner. Res 35(3):528–39 31805212 10.1002/jbmr.3911 PMC7184798

[91] Stapleton CJ, Pham MH, Attenello FJ, Hsieh PC. 2011. Ossification of the posterior longitudinal ligament: genetics and pathophysiology. Neurosurg. Focus 30(3):E6 10.3171/2010.12.FOCUS10271 21434822

[92] Epstein N. 2002. Ossification of the cervical posterior longitudinal ligament: a review. Neurosurg. Focus 13(2):1–10 10.3171/foc.2002.13.2.16 15916407

[93] Vaziri S, Lockney DT, Dru AB, Polifka AJ, Fox WC, Hoh DJ. 2019. Does ossification of the posterior longitudinal ligament progress after fusion? Neurospine 16(3):483–91 31607080 10.14245/ns.1938286.143 PMC6790726

[94] Chiba K, Ogawa Y, Ishii K, Takaishi H, Nakamura M, 2006. Long-term results of expansive open-door laminoplasty for cervical myelopathy—average 14-year follow-up study. Spine 31(26):2998–3005 17172996 10.1097/01.brs.0000250307.78987.6b

[95] Iwasaki M, Kawaguchi Y, Kimura T, Yonenobu K. 2002. Long-term results of expansive laminoplasty for ossification of the posterior longitudinal ligament of the cervical spine: more than 10 years follow up. J. Neurosurg 96(2 Suppl.):180–89 12450281

[96] Kalb S, Martirosyan NL, Perez-Orribo L, Kalani MY, Theodore N. 2011. Analysis of demographics, risk factors, clinical presentation, and surgical treatment modalities for the ossified posterior longitudinal ligament. Neurosurg. Focus 30(3):E11 10.3171/2010.12.FOCUS10265 21361749

[97] Mackenzie NC, Zhu D, Milne EM, van ’t Hof R, Martin A, 2012. Altered bone development and an increase in FGF-23 expression in *Enpp1*^−/−^ mice. PLOS ONE 7(2):e32177 22359666 10.1371/journal.pone.0032177 PMC3281127

[98] Babij P, Roudier M, Graves T, Han CY, Chhoa M, 2009. New variants in the *Enpp1* and *Ptpn6* genes cause low BMD, crystal-related arthropathy, and vascular calcification. J. Bone Miner. Res 24(9):1552–64 19419305 10.1359/jbmr.090417

[99] Anderson HC, Harmey D, Camacho NP, Garimella R, Sipe JB, 2005. Sustained osteomalacia of long bones despite major improvement in other hypophosphatasia-related mineral deficits in tissue non-specific alkaline phosphatase/nucleotide pyrophosphatase phosphodiesterase 1 double-deficient mice. Am. J. Pathol 166(6):1711–20 15920156 10.1016/S0002-9440(10)62481-9 PMC1602415

[100] Hajjawi MO, MacRae VE, Huesa C, Boyde A, Millan JL, 2014.Mineralisation of collagen rich soft tissues and osteocyte lacunae in *Enpp1*^−/−^ mice. Bone 69:139–47 25260930 10.1016/j.bone.2014.09.016 PMC4228085

[101] Nam HK, Liu J, Li Y, Kragor A, Hatch NE. 2011. Ectonucleotide pyrophosphatase/phosphodiesterase-1 (ENPP1) protein regulates osteoblast differentiation. J. Biol. Chem 286(45):39059–71 21930712 10.1074/jbc.M111.221689 PMC3234731

[102] Zimmerman K, Liu X, von Kroge S, Stabach P, Lester ER, 2022. Catalysis-independent ENPP1 protein signaling regulates mammalian bone mass. J. Bone Miner. Res 37(9):1733 —49 35773783 10.1002/jbmr.4640 PMC9709593

[103] Bodine PV, Zhao W, Kharode YP, Bex FJ, Lambert AJ, 2004. The Wnt antagonist secreted frizzled-related protein-1 is a negative regulator of trabecular bone formation in adult mice. Mol. Endocrinol 18(5):1222–37 14976225 10.1210/me.2003-0498

[104] Otero JE, Gottesman GS, McAlister WH, Mumm S, Madson KL, 2013. Severe skeletal toxicity from protracted etidronate therapy for generalized arterial calcification of infancy. J. Bone Miner. Res 28(2):419–30 22972716 10.1002/jbmr.1752

[105] Li Q, Guo H, Chou DW, Berndt A, Sundberg JP, Uitto J. 2013. Mutant *Enpp1^asj^* mice as a model for generalized arterial calcification of infancy. Dis. Model. Mech 6(5):1227–35 23798568 10.1242/dmm.012765 PMC3759342

[106] Albright RA, Stabach P, Cao W, Kavanagh D, Mullen I, 2015. ENPP1-Fc prevents mortality and vascular calcifications in rodent model of generalized arterial calcification of infancy. Nat. Commun 6:10006 26624227 10.1038/ncomms10006 PMC4686714

[107] Khan T, Sinkevicius KW, Vong S, Avakian A, Leavitt MC, 2018. ENPP1 enzyme replacement therapy improves blood pressure and cardiovascular function in a mouse model of generalized arterial calcification of infancy (GACI). Dis. Model. Mech 11(10):035691 10.1242/dmm.035691 PMC6215426 30158213

[108] Marrott PK, Newcombe KD, Becroft DM, Friedlander DH. 1984. Idiopathic infantile arterial calcification with survival to adult life. Pediatr. Cardiol 5(2):119–22 6473121 10.1007/BF02424963

[109] Thiaville A, Smets A, Clercx A, Perlmutter N. 1994. Idiopathic infantile arterial calcification: a surviving patient with renal artery stenosis. Pediatr. Radiol 24(7):506–8 7885787 10.1007/BF02015014

[110] Stabach PR, Zimmerman K, Adame A, Kavanagh D, Saeui CT, 2020. Improving the pharmacodynamics and in vivo activity of ENPP1-Fc through protein and glycosylation engineering. Clin. Transl. Sci 14(1):362–72 33064927 10.1111/cts.12887 PMC7877847

[111] Scannell JW, Blanckley A, Boldon H, Warrington B. 2012. Diagnosing the decline in pharmaceutical R&D efficiency. Nat. Rev. Drug. Discov 11(3):191–200 22378269 10.1038/nrd3681

[112] Gorzelany JA, de Souza MP. 2013. Protein replacement therapies for rare diseases: a breeze for regulatory approval? Sci. Transl. Med 5(178):178fs10 10.1126/scitranslmed.3005007 23536010

[113] Hay M, Thomas DW, Craighead JL, Economides C, Rosenthal J. 2014. Clinical development success rates for investigational drugs. Nat. Biotechnol 32(1):40–51 24406927 10.1038/nbt.2786

[114] Ringel MS, Scannell JW, Baedeker M, Schulze U. 2020. Breaking Eroom’s law. Nat. Rev. Drug Discov 19(12):833–34 32300238 10.1038/d41573-020-00059-3

[115] Cheng Z, O’Brien K, Howe J, Sullivan C, Schrier D, 2021. INZ-701 prevents ectopic tissue calcification and restores bone architecture and growth in ENPP1-deficient mice. J. Bone Miner Res 36(8):1594–604 33900645 10.1002/jbmr.4315

[116] Inozyme Pharma Inc. 2023. Inozyme Pharma reports topline data from ongoing phase 1/2 trials of INZ-701 GlobeNewswire, Feb. 16. https://www.globenewswire.com/news-release/2023/02/16/2609610/0/en/Inozyme-Pharma-Reports-Positive-Topline-Data-from-Ongoing-Phase-1-2-Trials-of-INZ-701.html

[117] Appelman-Dijkstra NM, Papapoulos SE. 2016. From disease to treatment: from rare skeletal disorders to treatments for osteoporosis. Endocrine 52(3):414–26 26892377 10.1007/s12020-016-0888-7 PMC4879160

[118] Hyder JA, Allison MA, Criqui MH, Wright CM. 2007. Association between systemic calcified atherosclerosis and bone density. Calcif. Tissue Int 80(5):301–6 17505774 10.1007/s00223-007-9004-6

[119] Farhat GN, Cauley JA, Matthews KA, Newman AB, Johnston J, 2006. Volumetric BMD and vascular calcification in middle-aged women: the Study of Women’s Health Across the Nation. J. Bone Miner Res 21(12):1839–46 17002567 10.1359/jbmr.060903

[120] Braun J, Oldendorf M, Moshage W, Heidler R, Zeitler E, Luft FC. 1996. Electron beam computed tomography in the evaluation of cardiac calcification in chronic dialysis patients. Am. J. Kidney Dis 27(3):394–401 8604709 10.1016/s0272-6386(96)90363-7

[121] Pimentel A, Urena-Torres P, Zillikens MC, Bover J, Cohen-Solal M. 2017. Fractures in patients with CKD—diagnosis, treatment, and prevention: a review by members of the European Calcified Tissue Society and the European Renal Association of Nephrology Dialysis and Transplantation. Kidney Int. 92(6):1343–55 28964571 10.1016/j.kint.2017.07.021

[122] Lomashvili KA, Khawandi W, O’Neill WC. 2005. Reduced plasma pyrophosphate levels in hemodialysis patients. J. Am. Soc. Nephrol 16(8):2495–500 15958726 10.1681/ASN.2004080694

[123] O’Neill WC, Sigrist MK, McIntyre CW 2010. Plasma pyrophosphate and vascular calcification in chronic kidney disease. Nephrol. Dial. Transplant 25(1):187–91 19633093 10.1093/ndt/gfp362 PMC4326300

[124] Collab. Comput. Proj. Number 4. 1994. The CCP4 suite: programs for protein crystallography. Acta Crystallogr. D Biol. Crystallogr 50(Part 5):760–3 15299374 10.1107/S0907444994003112

[125] Rupp T, Butscheidt S, Vettorazzi E, Oheim R, Barvencik F, 2019. High FGF23 levels are associated with impaired trabecular bone microarchitecture in patients with osteoporosis. Osteoporos. Int 30(8):1655–62 31044263 10.1007/s00198-019-04996-7

[126] Carrillo-Lopez N, Panizo S, Alonso-Montes C, Roman-Garcia P, Rodriguez I, 2016. Direct inhibition of osteoblastic Wnt pathway by fibroblast growth factor 23 contributes to bone loss in chronic kidney disease. Kidney Int. 90(1):77–89 27165819 10.1016/j.kint.2016.01.024

[127] Meyre D, Lecoeur C, Delplanque J, Francke S, Vatin V, 2004. A genome-wide scan for childhood obesity-associated traits in French families shows significant linkage on chromosome 6q22.31-q23.2. Diabetes 53(3):803–11 14988267 10.2337/diabetes.53.3.803

[128] Flanagan JM, Sheehan V, Linder H, Howard TA, Wang YD, 2013. Genetic mapping and exome sequencing identify 2 mutations associated with stroke protection in pediatric patients with sickle cell anemia. Blood 121(16):3237–45 23422753 10.1182/blood-2012-10-464156 PMC3630835

